# Navigating the landscape of PD-1/PD-L1 imaging tracers: from challenges to opportunities

**DOI:** 10.3389/fmed.2024.1401515

**Published:** 2024-06-07

**Authors:** Melinda Badenhorst, Albert D. Windhorst, Wissam Beaino

**Affiliations:** ^1^Amsterdam UMC location Vrije Universiteit Amsterdam, Department of Radiology and Nuclear Medicine, De Boelelaan, Amsterdam, Netherlands; ^2^Cancer Center Amsterdam, Imaging and Biomarkers, Amsterdam, Netherlands

**Keywords:** PD-L1, PD-1, PET imaging, tracer, immune checkpoint

## Abstract

Immunotherapy targeted to immune checkpoint inhibitors, such as the program cell death receptor (PD-1) and its ligand (PD-L1), has revolutionized cancer treatment. However, it is now well-known that PD-1/PD-L1 immunotherapy response is inconsistent among patients. The current challenge is to customize treatment regimens per patient, which could be possible if the PD-1/PD-L1 expression and dynamic landscape are known. With positron emission tomography (PET) imaging, it is possible to image these immune targets non-invasively and system-wide during therapy. A successful PET imaging tracer should meet specific criteria concerning target affinity, specificity, clearance rate and target-specific uptake, to name a few. The structural profile of such a tracer will define its properties and can be used to optimize tracers in development and design new ones. Currently, a range of PD-1/PD-L1-targeting PET tracers are available from different molecular categories that have shown impressive preclinical and clinical results, each with its own advantages and disadvantages. This review will provide an overview of current PET tracers targeting the PD-1/PD-L1 axis. Antibody, peptide, and antibody fragment tracers will be discussed with respect to their molecular characteristics and binding properties and ways to optimize them.

## 1 Introduction

Early studies investigating the roles of programmed cell death receptor 1 (PD-1) and its ligands (PD-L1 and PD-L2), could ascertain the immunomodulatory function of these proteins (1–4). PD-1 is primarily expressed on activated T and B cells, exhausted T cells, cytotoxic T cells, natural killer (NK) cells, monocytes, macrophages, dendritic cells (DCs) and myeloid progenitor cells (5). PD-L1 is constitutively expressed on T and B cells, macrophages, DCs, neutrophils and antigen-presenting cells (APCs), while expression of PD-L2 is mainly restricted to macrophages, DCs and resting B cells (6, 7). While both PD-L1 and PD-L2 play a role in immune regulation by interacting with PD-1, the exact function of PD-L2 (whether inhibitory or stimulatory) is currently still under debate (4, 7, 8). Interaction of PD-1 with PD-L1 causes a number of signaling events and cellular activities that aid in down-regulating cytotoxic immune responses and preventing an undesired and constantly activated immune system, thereby acting as immune checkpoint proteins (6, 9, 10). PD-L1 is overexpressed on tumor cells and the immunoregulatory interaction with PD-1 equips the tumor to successfully evade anti-tumor activity (6, 7, 11–13). The inability of the host’s immune system to distinguish between PD-L1 expression on normal cells and over-expression on tumor cells prevents T-cell-mediated cytotoxic killing and T-cell proliferation, while promoting T-cell apoptosis and increasing the number of exhausted T-cells (14). By inhibiting the interaction of PD-1 and PD-L1, this anti-tumor activity can effectively be restored and cytotoxic T cell killing of tumor cells can be initiated (15). Over the last couple of decades, PD-1/PD-L1 immune checkpoint inhibitors (ICIs) have been developed and tested in a myriad of cancer types with most exhibiting impressive clinical outcomes such as improved overall survival (OS), durable response and long-term clinical benefit, compared with conventional treatment approaches (6, 13, 16–21). Most of the ICIs currently approved by the Food and Drug Administration (FDA) are monoclonal antibodies (mAbs) that can bind to either PD-1 or PD-L1 with high specificity and affinity and block the interaction (12). Immunotherapy using mAbs has clearly demonstrated improved patient outcomes, however, not without some drawbacks. While they are considered more tolerable and less severe compared to conventional treatment, adverse events and potentially fatal treatment induced toxicities still occur in some patients when mAb immunotherapy is administered (22, 23). In addition, highly variable response rates among patients treated with PD-1/PD-L1 ICI have been reported, even showing no response at all or developing resistance to treatment after an initial positive response (24–26). The precise selection of patients that have the highest chance of optimal treatment benefit is still an unmet need (27, 28). Currently, efforts are focused on identifying and characterizing biomarkers that could predict treatment response (29). As an FDA-approved companion diagnostic method, assessment of PD-L1 status by invasive biopsies to perform immunohistochemistry (IHC) is the standard practice in the clinic (30). However, IHC is known to be a poor reflection of PD-L1 dynamic expression levels in both tumor and healthy tissue (31). Non-invasive detection of PD-1 or PD-L1 status using imaging techniques could be complementary to IHC and both preclinical and clinical trials have demonstrated its utility (32–34).

**FIGURE 1:**
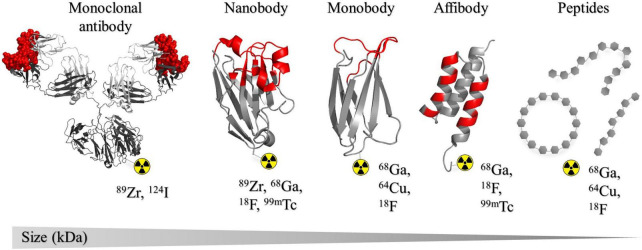
Structural representation of the different classes of binders that have been developed as radiotracers for PD-L1 and PD-1 discussed in this review. Structures are presented in order of size (kDa) large to small from left to right. The list of radioisotopes most commonly used in combination with each binder type is shown. Complementarity determining regions (CDRs) or binding regions are indicated in red. From left to right: murine IgG1 monoclonal antibody, PDB: 1IGY (243); KN035 nanobody, PDB: 5JDS (133); ySMB-9 monobody, PDB: 3RZW (244); Z_*HER*_2 affibody, PDB: 2KZJ (245). All crystal structures were created in PyMOL Molecular Graphics System, Version 1.2r3pre, Schrödinger, LLC.

Positron emission tomography (PET) tracers used for oncology are produced by combining a positron-emitting radioisotope with a targeting moiety such as full-length mAbs, antibody fragments, nanobodies, peptides, or small molecules (35). The choice of radioisotope is often dependent on the biological properties of the targeting moiety, such as size or *in vivo* half-life, and can range from radiohalogens (fluorine-18 and iodine-124) to radiometals (copper-64, gallium-68, and zirconium-89) (35, 36). In most cases, the physical half-life of the chosen radioisotope should closely match the *in vivo* biological half-life of the targeting molecule (35, 37). Non-invasive PET imaging using suitable tracers to track and assess the dynamic expression of PD-L1 and PD-1 has the potential to bring us one step closer to finding a more desirable response predictor, and could enable us to optimize those currently in use (36). Extensive preclinical and clinical research has been dedicated toward mAb-based PD-1/PD-L1 binders, and since there are already a number approved for clinical use, they were the obvious choice for initial development of PD-1/PD-L1-targeting imaging tracers (32). Notwithstanding the promising results from a number of PET imaging studies using mAbs, some intrinsic properties make them less suited as imaging tracers to assess the dynamics of PD-1/PD-L1 expression (38–42). For example, high-contrast images are usually obtained days after injection owing to their slow pharmacokinetics causing considerable background signal, ultimately leading to increased radiation dose (35). Additionally, their large size can limit tumor penetration ability, which can lead to an inaccurate assessment of the complete PD-L1/PD-1 expression landscape (34). The number of non-mAb PD-L1/PD-1 imaging tracers, including peptides, small molecules, and antibody fragments, are continuously growing in an attempt to overcome some of the shortcomings of full length mAbs (Figure 1). These imaging tracers can offer faster clearance, better tissue penetration and retention and lower production costs compared to mAbs (43, 44). Currently, efforts are focused on the development of a tracer that can accurately detect PD-1/PD-L1 expression levels in a shorter timeframe to allow repeated assessment in the same patient and better predict treatment response.

To fully optimize the properties of such a tracer, details about the molecular mode of action and structural interactions between the two binding partners are crucial (45–47). The molecular basis of the interaction of human PD-1 and PD-L1, in addition to the complex structures with the respective therapeutic mAbs of each, have already been solved by X-ray crystallography (46, 48, 49). Following these initial structural analysis studies, particular residues – defined as “hot-spots” – present in in PD-L1 and PD-1 have been identified, and interaction with these residues are shared among nearly each respective binder (48). Together, these studies revealed invaluable information about the binding mode on a molecular level of these immune checkpoint proteins. This in turn can aid in the design of new tracers and optimization of those already in development. For a more extensive overview of preclinical and clinical imaging results of PD-1 and PD-L1 imaging tracers the reader is referred to the following reviews: (32–34, 50, 51). In this review, an overview is provided of current PET tracers of different molecular classes targeting the PD-1/PD-L1 axis. Antibody, antibody fragment and peptide tracers will be discussed with respect to their current stage of development with special focus on their molecular characteristics and binding properties.

## 2 Monoclonal antibodies

Over the last three decades, mAbs have dominated protein-based drug therapy. By 2022, a total of 149 mAbs have either been approved or are in regulatory review by EU or US regulatory bodies (52). The success of mAbs can be attributed to their high affinity and specificity toward a target of interest. PD-1/PD-L1 ICI comprises a handful of FDA-approved mAbs that have led to impressive clinical benefits such as improved OS and durable progression-free survival (PFS) in cancer patients (13). Besides therapeutic application, there has been a shift toward utilizing these targeting mAbs for precisely this purpose using imaging techniques (see Table 1) (53).

**TABLE 1:** Current FDA-approved PD-L1 and PD-1 binding monoclonal antibodies and the developed antibody-based radiotracers.

Target	mAb (other names)	Commercial name	FDA approval year	Design strategy/screening technique	IgG subclass	Radiotracers	Affinity to human PD-L1/PD-1 (method)	Crystallization resolution
PD-L1	Durvalumab (MEDI14736)	Imfinzi^®^	2017	Hybridoma (60)	IgG1	[^89^Zr]Zr-DFO-durvalumab (65, 66) [^89^Zr]Zr-DFO-sq-durvalumab (40, 246)	Durvalumab: **IC_50_ = 0.1 nM** (HTRF) (60) Durvalumab-scFv: ***K*_D_ = 0.7 nM** (SPR) (61) Durvalumab: ***K*_D_ = 3.0 nM** (BLI) (198) Durvalumab: ***K*_D_ = 0.8 nM** (SPR) (247)	Durvalumab-scFc/hPD-L1*^a^*: **2.3 Å** (61) Durvalumab-Fab/hPD-L1*^b^*: **2.65 Å** (62)
	**Atezolizumab** (MPDL3280A)	Tecentriq^®^	2016	Phage display (67)	IgG1	[^89^Zr]Zr-DFO-atezolizumab (38, 73, 248)	Atezolizumab: ***K*_D_ = 0.4 nM** (SPR) (67) Atezolizumab-scFv: ***K*_D_ = 1.8 nM** (SPR) (61) Atezolizumab: ***K*_D_ = 2.3 nM** (BLI) (198) Atezolizumab: ***K*_D_ = 0.2 nM** (SPR) (247)	Atezolizumab/hPD-L1: **2.9 Å** (68) Atezolizumab-Fab/hPD-L1: **3.1 Å** (62)
	**Avelumab** (MSB0010718C)	Bavencio^®^	2017	Details not described	IgG1	[^89^Zr]Zr-DFO-avelumab (78)	Avelumab-scFv: ***K*_D_ = 42 pM** (SPR) (75) Avelumab-scFv**: *K*_D_ = 47 pM** (SPR) (61) Avelumab: ***K*_D_ = 4.9 nM** (BLI) (198) Avelumab: ***K*_D_ = 0.1 nM** (SPR) (247) ^89^Zr-DFO-avelumab: ***K*_D_ = 0.4 nM** (cellular assay) (78)	Avelumab-scFv/PD-L1: **3.2 Å** (75)
PD-1	**Nivolumab** (BMS-936558/MDX 1105)	Opdivo^®^	2014	Hybridoma (79)	IgG4	[^89^Zr]Zr-DFO-nivolumab (87)	Nivolumab: ***K*_D_ = 2.6 nM** (cellular assay), **3.1 nM** (SPR) and **2.7 pM** (BLI) (79) Nivolumab: ***K*_D_ = 1.5 nM** (SPR) (80) Nivolumab-scFv: **IC_50_ = 26 nM** (82)	Nivolumab-Fab/hPD-1: **2.4 Å** (80)
	**Pembrolizumab** (MK-3475)	Keytruda^®^	2014	Details not described	IgG4	[^89^Zr]Zr-DFO-pembrolizumab (98) [^64^Cu]Cu-DOTA-pembrolizumab (96)	Pembrolizumab: ***K*_D_ = 27 pM** (ELISA) (90) Pembrolizumab: ***K*_D_ = 29 pM** (solid phase interferometry), **120 pM** (SPR), **130 pM** (SPR), **51 pM** (SPR), and **1.1 pM** (kinetic exclusion assay) (249)	Pembrolizumab-Fab/hPD-1: **2.9 Å** (90)
	**Cemiplimab** (REGN2810)	Libtayo^®^	2018	Hybridoma (100)	IgG4	n.a.*^c^*	Cemiplimab: ***K*_D_ = 6.1 nM** for monomeric PD-1 and **0.6 nM** for dimeric PD-1 (SPR) (100) Cemiplimab: ***K*_D_ = 1.7 nM** (SPR) (107)	Cemiplimab-scFv/hPD-1: **3.4 Å** (107) Cemiplimab-Fab/hPD-1: **1.98 Å** (104)
	**Dostarlimab** (TSR-042)	Jemperli^®^	2021	Hybridoma (250)	IgG4	n.a.	Dostarlimab: ***K*_D_ = 0.3 nM** (SPR) (250)	Dostarlimab-Fab/hPD-1: **1.53 Å** (105)
	**Retifanlimab** (INCMGA00012)	Zynyz^®^	2023	Details not described	IgG4	n.a.	Retifanlimab: ***K*_D_ = 0.6 nM** (SPR) (108)	n.d.^d^
	**Toripalimab** (JS001)	Loqtorzi™	2023	Details not described	IgG4	[^99m^Tc]Tc-toripalimab (113) [^124^I]I-toripalimab (114, 115)	Toripalimab: ***K*_D_ = 3.8 nM** (cellular assay) (251) Toripalimab: ***K*_D_ = 0.3 nM** (cellular assay) (106) [^125^I]I-toripalimab: ***K*_D_ = 4.3 nM** (cellular assay) (114)	Toripalimab-Fab/hPD-1: **2.6 Å** (106)

^a^scFv, single-chain fragment variable; ^b^Fab, fragment antigen-binding; ^c^Not applicable; ^d^Not determined. These are the affinity values (in nanomolar) and crystallization resolution (in Ångström), they are written in bold for emphasis and quick identification.

Immuno-PET combines radiolabeled mAbs and the sensitivity of the PET imaging technique (54) and with immuno-PET, the application of mAbs is further expanded to determine drug pharmacokinetics (PK) and pharmacodynamics (PD) parameters such as optimal drug dosage and biodistribution (55). Moreover, assessing dynamic changes and heterogeneity of target expression non-invasively in different organs is also made possible with immuno-PET (55). This information is crucial for the successful stratification of patients and developing customized treatment approaches to better predict therapeutic response. In recent years, ^89^Zr-immuno-PET has been established as a valuable tool for obtaining such information within clinical practice and academic research (37). Zirconium-89 is a positron emitter with a half-life (*t*_1/2_ = 78.4 h) that conveniently matches the biological half-life of intact long-circulating therapeutic mAbs and makes distribution from the cyclotron to required national or international destinations logistically feasible. Chelation of zirconium-89 with commercially available bifunctional chelators following good manufacturing practice (GMP) has also been established and allows for effortless radiolabeling (56). Other radioisotopes, for example, copper-64 (*t*_1/2_ = 12.7 h) and iodine-124 (*t*_1/2_ = 100.2 h), have also been used for immuno-PET applications (57), however, zirconium-89 is used more extensively in preclinical and clinical studies due to its lower cost, wider availability, and suitability for radiolabeling mAbs (37, 58, 59).

### 2.1 PD-L1: durvalumab

Durvalumab was first produced using hybridoma technology and immunization of IgG2 and IgG4 XenoMouse mice models (60). Thereafter, the constant domain of the Ab was substituted for a human IgG1 domain with a mutated Fc region that leads to reduced antibody dependent cellular cytotoxicity (ADCC) and complement-dependent cytotoxicity (CDC) (60). The same group reported an IC_50_ value of 0.1 nmol/L in a competition assay with PD-1. Tan et al. (61) demonstrated a high binding affinity of the single chain Fv fragment of durvalumab toward human PD-L1 by SPR and measured a *K*_D_ of 0.667 nM. At these earlier timepoints of development, little information was known about the binding mechanism and interaction surface of these emerging mAbs. Lee et al. (62) reported some of the first co-crystallization results of PD-L1 with PD-1 and anti-PD-L1 mAbs, including durvalumab. Sixteen amino acid residues of PD-L1, primarily located within the central CC′FG β-sheet, CC′loop and N-terminal region, are responsible for the high affinity interaction of durvalumab (62). It was further shown that the variable domain of the heavy chain (VH) and light chain (VL) of durvalumab contribute to PD-L1 binding (61). Since its approval by the FDA in 2017, durvalumab has been a key role player in the development of immunotherapy toward improving patient outcomes, and results from clinical trials have been encouraging (16). Durvalumab has led to improved major pathological response (MPR) and disease-free survival (DFS) when administered as neoadjuvant and maintenance treatment for resectable and unresectable stage III non-small cell lung cancer (NSCLC) patients, respectively (63, 64). However, the clinical trial for resectable NSCLC patients receiving neoadjuvant durvalumab highlighted a need for better patient selection since an unexpectedly high mortality related to cardiovascular and respiratory comorbidities resulted in premature termination of the trial (63). Recent clinical PET-imaging studies have been initiated to test the feasibility and safety of using [^89^Zr]Zr-DFO-durvalumab for imaging PD-L1 expression. In a study by Smit et al. (65) PET/CT results were compared for patients receiving [^89^Zr]Zr-DFO-durvalumab only and patients receiving a 750 mg unlabeled durvalumab co-injection to reduce the tracer sink effect. However, due to the occupation of PD-L1 by a much higher dose of therapeutic durvalumab in the latter condition, overall tracer uptake was decreased. Furthermore, while they could show that [^89^Zr]Zr-DFO-durvalumab PET/CT was well-tolerated in patients with NSCLC, they were unable to demonstrate a significant correlation between patient response and tracer uptake in lesions (65). Similarly, [^89^Zr]Zr-DFO-durvalumab PET imaging proved well-tolerated in patients with squamous cell carcinoma of the head and neck (SCCHN) (66). In line with the study by Smit et al. (65) treatment response was also not correlated with tumor uptake or PD-L1 expression (66).

### 2.2 PD-L1: atezolizumab

Atezolizumab was identified using phage display technology by screening phage libraries expressing human VL and VH against human and murine PD-L1 (67). The clone selected and developed to become atezolizumab bound both human and mouse PD-L1 with high affinities as measured by SPR (*K*_D_ = 0.2 and 0.6 nM, respectively) (67). Affinities measured with binding assays on recombinant human and mouse PD-L1-expressing HEK293 cells were reported as *K*_D_ = 0.4 and 0.1 nM, respectively (67). In the study performed by Lee et al. (62), structural details about the interaction of atezolizumab and PD-L1 were elucidated as well. Twenty-three amino acid residues in the central CC′FG β-sheet, BC, CC′, C′C′′ and FG loops are responsible for the tight interaction with atezolizumab (62). By alanine scanning studies, Zhang et al. (68) identified Glu58 and Arg113 of PD-L1 as two major contributors to the high affinity of atezolizumab. Interestingly, while PD-L1 backbone conformation remains rigid upon binding to anti-PD-L1 mAbs, only atezolizumab binding induces a slight change in the PD-L1 BC loop to allow more interactions with the mAb (62). Atezolizumab was the first FDA-approved PD-L1 inhibitor for the treatment of cancers patients with urothelial carcinoma, metastatic NSCLC and triple-negative breast cancer (TNBC) (69–71). Given the favorable results from the IMpower010 clinical trial (NCT02486718, Impower010), atezolizumab has recently been approved as adjuvant therapy following surgery and chemotherapy in patients with stage II to IIIA NSCLC based on PD-L1 expression score using the Ventana PD-L1 assay (SP263, Ventana Medical Systems) as companion diagnostic (72). As with other PD-L1 blocking mAbs, the therapy response rate is difficult to predict. Bensch et al. (73) performed the first-in-human PET imaging study using [^89^Zr]Zr-DFO-atezolizumab to test feasibility and response prediction in metastatic urothelial carcinoma, NSCLC, and TNBC. Contrary to [^89^Zr]Zr-DFO-durvalumab, this study reported that better tumor response correlated well with increased tumor tracer uptake, while PD-L1 IHC expression gave no indication of such a correlation (73). Currently, a phase II diagnostic imaging trial is underway to evaluate whether [^89^Zr]Zr-DFO-atezolizumab PET/CT imaging can be used as a predictive tool to select patients with metastatic TNBC to receive PD-L1 inhibitors in addition to chemotherapy (74).

### 2.3 PD-L1: avelumab

Unlike durvalumab and atezolizumab, avelumab is a human IgG1 designed without a modified Fc region and can therefore mediate ADCC (75). While the binding affinities of durvalumab and atezolizumab are somewhat comparable, avelumab binds human PD-L1 in the picomolar range (*K*_D_ = 47 and 42 nM, measured by SPR independently) (61, 75). Crystal structures of the PD-L1-avelumab complex have revealed that even though both VL and VH are involved, the interaction with PD-L1 is dominated by the VH of avelumab (75). Additionally, the C strand, C′ strand, F strand, G strand, and CC′ loop present in PD-L1 are primarily involved in interaction (75). Avelumab is a promising treatment option for advanced and metastatic urothelial carcinoma (76, 77). Results from a recent phase III clinical trial (NCT02603432) have demonstrated that OS at 1 year was significantly longer in patients receiving avelumab as maintenance treatment (71.3%) compared to the control group who received best supportive care alone (58.4%) (76). In addition, the patient population with PD-L1-positive tumors vs. PD-L1-negative tumors determined by the Ventana PD-L1 assay (SP263, Ventana Medical Systems), which qualitatively detects PD-L1 expression in histology tissues, showed a significant increase in OS, further supporting the requirement for assessment of PD-L1 status (76). Studies to assess PD-L1 expression in humans using non-invasive PET imaging and radiolabeled avelumab have been limited. Zirconium-89-labeled avelumab has been investigated primarily in preclinical studies in which high specificity and affinity to PD-L1 were demonstrated on PD-L1-positive MDA-MB-231 human breast adenocarcinoma cells (78). In addition, [^89^Zr]Zr-DFO-avelumab was investigated in mice models bearing MDA-MB-231 tumors and could show the feasibility of imaging PD-L1 with this tracer (39, 78). A phase I clinical trial is currently underway to evaluate the feasibility of using [^89^Zr]Zr-DFO-avelumab to assess PD-L1 expression in patients with NSCLC and whether prediction of avelumab treatment response is possible (NCT03514719, PINNACLE).

### 2.4 PD-1: nivolumab

Nivolumab, developed and characterized by Bristol-Myers Squibb (BMS), is an anti-PD-1 mAb that was produced in humanized mice using hybridoma technology (79). The lead antibody clone was grafted onto a human IgG4 bearing an S228P mutation for increased stability and reduced ADCC and CDC activity (79). *In vitro* characterization showed that nivolumab could bind PD-1-expressing CHO cells and activated T cells with EC_50_ = 1.7 and 0.6 nM, respectively (79). The crystal structures of nivolumab in complex with PD-1 have been studied extensively to gain more insight into the PD-1 epitope and molecular mode of inhibition (80–82). Tan et al. (80) solved the crystal structures of this complex and showed that both VH and VL of nivolumab are involved in binding. Residues present in the FG and BC loops of the IgV domain and the N-loop of PD-1 are responsible for interaction with nivolumab (80). Interestingly, most of the hydrogen bonds (10 of 16) are formed within the N-loop, making it the most dominant interacting region of nivolumab (80). This study further confirmed that, unlike previously speculated, N-glycosylation is not required for nivolumab binding (80). Results from clinical trials in recent years have shown encouraging results for a multitude of cancers, such as NSCLC, melanoma, and esophageal squamous-cell carcinoma, to name a few (21, 83–85). Compared to a chemotherapy treatment, docetaxel, nivolumab led to significant improvement in OS, objective response rate (ORR) and overall tolerability in NSCLC (83). It has also been shown that nivolumab as adjuvant therapy after resection in urothelial carcinoma and melanoma patients resulted in overall prolonged DFS and recurrence-free survival (RFS), respectively (21, 86). While treatment outcomes are promising, these studies also highlighted the current lack of methods to predict ICI response accurately. In a preclinical PET imaging study to determine tracer clearance and biodistribution in healthy cynomolgus monkeys, nivolumab was conjugated to DFO and subsequently labeled with zirconium-89 (87). [^89^Zr]Zr-DFO-nivolumab uptake was visualized clearly in the spleen, an organ with distinct populations of PD-1-expressing DCs, as well as in the liver, evident of mAb catabolism (87). England et al. (88) further demonstrated in another preclinical study the feasibility and efficiency of imaging PD-1 on T cells in a humanized lung cancer mouse model with [^89^Zr]Zr-DFO-nivolumab. Niemeijer et al. (41) performed the first-in-human PET imaging study using [^89^Zr]Zr-DFO-nivolumab in patients with NSCLC. Similar to the study in cynomolgus monkeys, higher [^89^Zr]Zr-DFO-nivolumab uptake was visualized in the spleen and the liver compared to other organs (41). A positive correlation between nivolumab treatment response at 3 months and [^89^Zr]Zr-DFO-nivolumab peak standard uptake value (SUV_peak_) at baseline prior to treatment could be drawn, however, the sample population for this study was small (13 patients) (41).

### 2.5 PD-1: pembrolizumab

Merck & Co., Inc. developed pembrolizumab, an anti-PD-1 mAb able to block the PD-1/PD-L1 interaction with high affinity (*K_D_* ≈ 29 pM) (89). Similar to nivolumab, pembrolizumab is a humanized mAb that is also based on a human IgG4 antibody containing the S228P mutation (89). Na et al. (90) investigated the molecular mode of action by solving the crystal structures of the human PD-L1-pembrolizumab complex. While the complementarity-determining regions (CDRs) and framework region (FR) of pembrolizumab are involved in the interaction, two key regions within PD-1 are responsible for contact (90). The first region consists of residues present in the C′D loop, and the second region consists of residues present in the C, C′, and F strands of PD-1 (90). The structural analysis performed by Tan et al. (80) also confirmed that while the interaction surfaces are close, there are no overlapping regions between nivolumab and pembrolizumab on the surface of human PD-1. The last decade has shown impressive clinical results for pembrolizumab as first-line treatment, monotherapy and neoadjuvant therapy for patients with PD-L1-expressing NSCLC, melanoma, and advanced gastric cancer who benefit from prolonged OS, manageable side effects and durable responses (91–95). A couple of preclinical studies have evaluated radiolabeled pembrolizumab in rodents (96, 97). England et al. (97) showed that pembrolizumab modified with p-SCN-deferoxamine and radiolabeled with zirconium-89 could accumulate in the salivary glands (containing PD-1-expressing T-cells) of humanized NSG mice engrafted with human peripheral blood mononuclear cells (PBMCs). In a similar study, Natarajan et al. (96) imaged PD-1 expression on tumor infiltrating lymphocytes (TILs) in humanized NSG mice bearing A375 human skin melanoma tumors with ^89^Zr- and ^64^Cu-labeled pembrolizumab. For both tracers, uptake in the tumor and spleen could be visualized, indicative of PD-1 detection (96). Another preclinical study was performed with healthy cynomolgus monkeys to better evaluate the potential clinical translation of radiolabeled pembrolizumab for tracking PD-1 expression (98). In this study, uptake of the tracer (pembrolizumab conjugated to N-suc-desferal-TFP ester and radiolabeled with zirconium-89) was visible in the expected lymphoid organs such as the spleen, lymph nodes, and tonsils (98). Clinical PET imaging with [^89^Zr]-DFO-pembrolizumab in patients with NSCLC was able to detect tumor lesions and, notably, response to anti-PD-1 treatment could be correlated with higher [^89^Zr]Zr-DFO-pembrolizumab uptake (42, 99).

### 2.6 PD-1: emerging mAbs

While the therapeutic mAbs described above have made great strides in the field of immunotherapy, new mAbs are continuously being developed. A couple of emerging anti-PD-1 mAbs that have gained FDA approval in more recent years include cemiplimab (2018), dostarlimab (2021), retifanlimab (2023), and toripalimab (2023) (52). Similar to nivolumab and pembrolizumab, all are humanized mAbs based on human IgG4 (100–103). Structural analysis studies have revealed that the binding interface of cemiplimab and dostarlimab with PD-1 are highly similar and include the BC, C′D and FG loops present in PD-1 (104, 105). Interaction with toripalimab was shown to be dominated by the FG loop of PD-1, and binding was independent of glycosylation (106), while glycosylation of N58 in PD-1 improved the affinity of cemiplimab substantially (107). Similar to the other two anti-PD-1 mAbs, cemiplimab, dostarlimab, retifanlimab, and toripalimab can bind human PD-1 with high affinity as measured by SPR (*K*_D_ = 6.1, 0.3, 0.6, and 0.2 nM, respectively) (100, 101, 106, 108). Various studies have shown the clinical benefit and durable responses following treatment with these mAbs as monotherapy or combined with chemotherapeutic agents, and many clinical trials are still ongoing (103, 109–112). Considering the development and clinical assessment of these recently approved mAbs are still in the early stages, the number of imaging studies performed to date has been limited. Thus far, toripalimab is the only candidate from this group that have been explored as a PET (and SPECT) imaging tracer (113–115). Huang et al. (114) radiolabeled toripalimab with iodine-124 and evaluated the tracer in humanized PD-1-C57BL/6 mice bearing mouse sarcoma S180 tumors. [^124^I]I-toripalimab uptake could be visualized in tumors and the highest tumor-to-organ ratios were obtained at 72 h p.i. (114). The encouraging results from the preclinical study prompted the first-in-human pilot clinical translation study by the same group (115). Twelve patients, having either melanoma or urologic cancer, were administered a single dose of [^124^I]I-toripalimab (115). The tracer was well tolerated and proven safe in all patients, and peak uptake in tumor lesions was visualized 24 h p.i. (115). In addition, PET/MR was the preferred imaging modality over PET/CT for the detection of lesions located in the liver (115).

## 3 Non-antibody binders

Advances in recombinant antibody technology have made it possible to manipulate non-immunoglobulin or immunoglobulin-like architectures into high-affinity binding proteins, known as engineered protein scaffolds (116). Characteristically, these scaffolds are rigid, single-chain protein structures that are thermodynamically stable, soluble, contain conserved FRs, and have modifiable sequence diversity within variable binding regions (116, 117). Using directed evolution, these variable binding regions are subjected to site-directed mutagenesis and selection to generate randomized, highly complex and diverse combinatorial libraries (118). These libraries are then cloned into display vectors such as phages, yeast cells, bacterial cells, ribosomes, or messenger RNA (119–122). To identify high-affinity binders, the naïve combinatorial libraries are screened against a particular immobilized target of interest by displaying the engineered protein scaffold to the prescribed target (119). Examples of protein scaffolds that have gained considerable success in delivering high-affinity target binders include 10th fibronectin type III domain-based Adnectins (10Fn3) or monobodies, camelid heavy chain-only immunoglobulin derived nanobodies (Nbs) and helix-bundle proteins derived from *Staphylococcus aureus* protein A or affibodies, to name a few (see Table 2) (57, 123, 124).

**TABLE 2:** Programmed cell death ligand 1 and PD-1 binding nanobodies, monobodies, and affibodies in development as radiotracers.

Target	Type	Binder (other names)	Size (kDa)	Design strategy/screening technique	Radiotracers	Affinity to human PD-L1/PD-1 (method)	Development stage	Docking/ crystallization
PD-L1	Nanobody	**KN035** (Envafolimab)	79.6	Camel immunization and phage display (133)	[^89^Zr]Zr-DFO-KN035 (135) [^99m^Tc]Tc-HYNIC-KN035 (136)	KN035: ***K*_D_ = 3.0 nM** (BLI) (133) KN035: ***K*_D_ = 2.9 nM** (BLI) [^99m^Tc]Tc-HYNIC-KN035: ***K*_D_ = 31 nM** (cellular assay) (136)	Clinical (137)	Crystallization (133)
		**Nb109**	14	Details not described	[^68^Ga]Ga-NOTA-Nb109 (141–144) [^68^Ga]Ga-NODA-CDV-Nb109 (145) [^131^I]I-Nb109 (146)	Nb109: ***K*_D_ = 2.9 nM** (SPR) (141) [^68^Ga]Ga-NODA-CDV-Nb109: ***K*_D_ = 12 nM** (cellular assay) (145)	Preclinical	n.d.^a^
		**NM-01**	14.3	Camel immunization and phage display (147)	[^99m^Tc]Tc-tricarbonyl-NM-01 (147–149, 252)	NM-01: ***K*_D_ = 1.8 nM** (ELISA) and **0.8 nM** (SPR) (147)	Clinical (147–149, 252)	n.d.
		**K2** (hPD-L1 Nb)	15	Alpaca immunization and phage display (150)	[^99m^Tc]Tc-tricarbonyl -K2 (150) [^68^Ga]Ga-NOTA-K2 (151) [^68^Ga]Ga-NOTA-mal-K2 (152)	K2: ***K*_D_ = 3.8 nM** (SPR) (150) K2-NOTA: ***K*_D_ = 3.7 nM** (SPR) (151) [^68^Ga]Ga-NOTA-K2: ***K*_D_ = 0.8 nM** (cellular assay) (151) K2: ***K*_D_ = 2.1 nM** (SPR) (152) NOTA-mal-K2: ***K*_D_ = 4.4 nM** (SPR) (152)	Preclinical	n.d.
		**APN09**		Details not described	[^68^Ga]Ga-THP-APN09 (153)	[^68^Ga]Ga-THP-APN09: ***K*_D_ = 22 nM** (cellular assay) (153)	Preclinical	n.d.
	Monobody (Adnectin)	**BMS-986192**	10	mRNA display with a 10Fn3 combinatorial library (158)	^18^F-BMS-986192 (41, 158, 159, 253) [^68^Ga]Ga-DOTA-BMS-986192 (160) [^68^Ga]Ga-NODAGA-BMS-986192 (162)	BMS-986192: ***K*_D_ < 10 pM** (SPR) (158) ^19^F-BMS-986192: ***K*_D_ < 10 pM** (SPR) (158) [^68^Ga]Ga-DOTA-BMS-986192: **IC_50_ = 2 nM** (cellular assay) (160) [^68^Ga]Ga-NODAGA-BMS-986192: **IC_50_ = 8.9 nM** (cellular assay) (162)	Clinical (41, 161, 253)	n.d.
		**FN3_hPD–L1–01_**	12	Yeast display with a 10Fn3 combinatorial library (164)	n.a.^b^	FN3_hPD–L1–01_: ***K*_D_ = 2.4 nM** (cellular assay) (164)	Preclinical	n.d.
		**FN3_hPD–L1_**	12	Yeast display with a 10Fn3 combinatorial library (163)	[^64^Cu]Cu-DOTA- FN3_hPD–L1_ (163)	FN3_hPD–L1_: ***K_D_* = 0.6 nM** (BLI) (163) FN3_hPD–L1_: ***K_D_* = 1.4 nM** (cellular assay) (163)	Preclinical	n.d.
	Affibody	**Z_PD–L1_1_**	7	Details not described	Al[^18^F]F-NOTA-Z_PD–L1_1_ (178, 179) [^68^Ga]Ga-NOTA-Z_PD–L1_1_ (179)	NOTA-Z_PD–L1_1_: ***K*_D_ = 1.3 nM** (SPR) (178) Al[^18^F]F-NOTA-Z_PD–L1_1_: ***K*_D_ = 70 pM** (cellular assay) (179)	Preclinical	n.d.
		**Z_PD–L1_4_**	7	Details not described	Al[^18^F]F-NOTA-Z_PD–L1_4_ (174) [^68^Ga]Ga-NOTA-Z_PD–L1_4_ (174)	NOTA-Z_PD–L1_4_: ***K*_D_ = 70 pM** (SPR) (174)	Preclinical	n.d.
		**PDA**	8	Details not described	[^99m^Tc]Tc-PDA (180)	[^99m^Tc]Tc-PDA: ***K*_D_ = 10 nM** (180)	Preclinical	n.d.
		**Z-j1 and Z-j2**	7	Yeast two hybrid library screening (181)	n.a.	n.d.	*In vitro* characterization	n.d.
PD-1	Nanobody	**PD-1-Nb20**	13.4	Camel immunization and phage display (154)	n.a.	PD-1 Nb20: ***K*_D_ = 179 pM** (SPR) (154)	Preclinical	n.d.
		**Anti-PD-1 Nb-Fc**	40	Phage display (155)	n.a.	Anti-PD-1 Nb-Fc: ***K*_D_ = 6.6 nM** (SPR) (155)	Preclinical	n.d.

^a^Not determined; ^b^Not applicable. These are the affinity values (in nanomolar) and crystallization resolution (in Ångström), they are written in bold for emphasis and quick identification.

### 3.1 Nanobodies

Since their serendipitous discovery 30 years ago, Nbs have been at the forefront of both treatment and diagnosis of a multitude of human diseases due to their unique and favorable biophysical properties (125, 126). Nbs are uniquely present in the serum of mammals belonging to the Camelidae family and are often referred to as single-domain antibodies or VHH because they are composed of only the variable (V)-domain of the heavy (H)-chain of a conventional IgG (124). Their small size of 15 kDa, well below the cutoff for glomerular filtration by the kidneys (∼50 kDa), makes Nbs suitable for applications requiring rapid tissue penetration or blood clearance, such as targeted drug delivery and imaging (127, 128). Another striking feature of Nbs is their excellent *in vivo* stability. This could be explained by the replacement of highly conserved and hydrophobic amino acids in the VH that would usually interact with the VL (for conventional immunoglobulins) with amino acids that are either smaller in size or are more hydrophilic (124, 129). Another attractive characteristic of Nbs is low immunogenicity, which is an important requirement for the clinical implementation of any pharmaceutical (130). Similar to a VH of a conventional Ab, three CDRs are present in Nbs. However, differences such as a longer H3 loop (so-called because of its position on CDR3 of a H-chain variable region), higher sequence conservation and solubility-enhancing mutations present in the FR, contribute to the high specificity and affinity (nM to pM) achieved by Nbs (131). Taken together, these properties are ideal for developing Nbs as PET imaging tracers. A couple of promising Nbs targeting the PD-1/PD-L1 axis have already been identified and are currently being investigated (132).

#### 3.1.1 PD-L1: KN035

Recognizing the potential of Nbs, a group of researchers at Alphamab Oncology immunized camels with human PD-L1 and constructed a Nb library that was screened by phage display to identify the most promising binder (133). They identified a Nb and fused it with human IgG1 Fc protein to produce KN035. Confirmed by biolayer interferometry (BLI) and competitive ELISA, KN035 binds with high affinity (*K*_D_ = 3.0 nM) to PD-L1 and strongly inhibits the PD-1/PD-L1 interaction (IC_50_ = 5.3 nM), respectively (133). The crystal structures of KN035, and in complex with PD-L1, revealed important mechanistic information about the specific residues involved in the interaction (133). Loop H3, and to a lesser extent loop H1, of KN035 are primarily responsible for the tight interaction with the flat binding surface on PD-L1. In combination, these loops form a binding surface consisting of a hydrophobic region enclosed by hydrophilic residues that take part in strong hydrogen and ionic bonds with residues present in PD-L1 (133). Interestingly, alanine-scanning and mutagenesis studies further identified five hot spot residues in PD-L1 critical for binding with KN035 (130). Alanine substitution of Tyr56, Ile54, Arg113, Glu58, and Gln66 in PD-L1 decreased the binding affinity with KN035 400-, 80-. 178-, 50-, and 162-fold, respectively, suggesting these residues are critical for binding (133). This is in agreement with previously identified human PD-L1 hot spot residues identified in co-crystalized structures of PD-L1 in complex with mAbs and encompasses residues present in the PD-1/PD-L1 binding interface (134). A strong anti-tumor effect was achieved in an A375-PD-L1 xenograft tumor model when treated with KN035 (133). KN035 was further evaluated as a PET imaging tracer by labeling it with zirconium-89 (135). [^89^Zr]Zr-Df-KN035 was injected into BALB/c nude mice bearing human glioma tumors (LN229 xenografts) and healthy cynomolgus monkeys (non-human primates, or NHP) to evaluate tumor uptake and tracer biodistribution, respectively (135). In the glioma tumor model, high [^89^Zr]Zr-Df-KN035 tumor uptake was achieved as soon as 24 h p.i. and could be retained up to 120 h p.i., while uptake in bone tissue increased notably at later time points of (72–120 h p.i.) (135). Biodistribution results in the NHP revealed high accumulation in the liver, kidneys, and gall bladder (135). Another group developed a SPECT imaging tracer using KN035 (136). The radiolabeled version, [^99m^Tc]Tc-HYNIC-KN035, retained its high affinity to PD-L1 (*K*_D_ = 31 nM) as determined by cellular saturation binding assays (136). [^99m^Tc]Tc-HYNIC-KN035 was injected into BALB/c nude mice bearing H1975 tumors (136) that could be visualized as soon as 4 h p.i. (136). These two studies clearly showcased the potential of KN035 as an imaging tracer. In addition, KN035 (now also known as Envafolimab) holds significant promise as a therapeutic agent and already entered multiple clinical trials not long after its discovery (137–139). Envafolimab is the first PD-L1 inhibitor that can be administered subcutaneously and has already been designated orphan drug status by the FDA for biliary tract cancer and soft tissue sarcoma (140).

#### 3.1.2 PD-L1: Nb109

This Nb was discovered and evaluated as a ^68^Ga-labeled PET tracer by Lv et al. (141). SPR confirmed high-affinity binding of Nb109 to PD-L1 (*K*_D_ = 2.9 nM) and, unlike most of the PD-L1 binders discussed in this review, competitive binding studies revealed binding of Nb109 to a different epitope compared to PD-1 and an anti-PD-L1 antibody (141). [^68^Ga]Ga-NOTA-Nb109 was prepared by conjugating NOTA to a Lys residue in Nb109 followed by radiolabeling with gallium-68 (141). Cell binding studies demonstrated good [^68^Ga]Ga-NOTA-Nb109 uptake in A375-hPD-L1 cells both untreated and treated with 1,000-fold KN035, further confirming the ability of Nb109 to bind to a separate binding site (141). In A375-hPD-L1 tumor-bearing mice, rapid tumor uptake and blood clearance were achieved, yielding high-contrast images as early as 1 h p.i. (141). In addition to quick visualization of PD-L1-expressing tumors, Nb109 proved useful for specifically monitoring the dynamic changes in expression levels of PD-L1 induced in tumor cells following chemotherapy treatment such as cisplatin, 5-fluorouracil and oxaliplatin (142, 143). In addition to a PD-L1-transfected tumor cell line and endogenous PD-L1-expressing tumor cell lines, [^68^Ga]Ga-NOTA-Nb109 could also successfully detect expression in patient-derived xenograft lung cancer tumors (144). To further optimize the signal-to-noise ratio of this tracer, Chen et al. (145) incorporated a tripeptide (Cys-Asp-Val or CDV) into the tail region of the Nb109 sequence to enable site-specific chelation with NODA and subsequent radiolabeling with gallium-68. [^68^Ga]Ga-NODA-CDV-Nb109 demonstrated excellent stability *in vitro* and *in vivo*, and A375-hPD-L1 cell binding studies demonstrated no considerable change in affinity (*K*_D_ = 12 nM) (145). All the imaging studies performed using this tracer showed that rapid and retained tumor uptake could be achieved, clearance occurs in the kidneys, and uptake in non-specific organs was low (141–145). In a recent study, Zhu et al. (146) labeled Nb109 with iodine-131 and explored its therapeutic effect on PD-L1-positive NSCLC tumors *in vivo*. Killing of tumor cells and increased tumor immunogenicity was achieved upon direct intratumoral injection of [^131^I]I-Nb109 in H460 tumor-bearing mice (146). Taken together, results from these studies clearly demonstrate the potential of Nb109 as both a diagnostic and therapeutic agent.

#### 3.1.3 PD-L1: NM-01

Immunization of camels with the extracellular domain of PD-L1 and subsequent selection by phage display allowed the identification of a Nb, NM-01, designed with a C-terminal His-tag, and unique PD-L1 binding properties (147). NM-01 could bind recombinant PD-L1 with high affinity as measured by both ELISA and SPR (*K*_D_ = 1.8 and 0.8 nM, respectively) (147). Interestingly, and similar to Nb109, NM-01 did not block the interaction of PD-L1 with either PD-1 or atezolizumab, indicating a separate binding epitope (147). NM-01 was produced in compliance with GMP standards, radiolabeled with technetium-99m, and evaluated *in vivo* in mice bearing HCC827 tumors (147). Due to rapid clearance by the kidneys, high-contrast images could be acquired as early as 30 min p.i. and [^99m^Tc]Tc-tricarbonyl-NM-01 tumor uptake was retained up to 1.5 h p.i. (147). Similar uptake was observed in mice pre-dosed with atezolizumab 4 days before [^99m^Tc]Tc-tricarbonyl-NM-01 injection, further validating that this Nb binds to a different epitope (147). An early phase I study using [^99m^Tc]Tc-tricarbonyl-NM-01 in patients with NSCLC demonstrated its safety and ability to track primary tumors and metastases as early as 2 h p.i (148). In a clinical study investigating myocarditis, which is known to be associated with ICI therapy, [^99m^Tc]Tc-tricarbonyl-NM-01 could successfully assess PD-L1 expression in the myocardium of NSCLC patients about to receive ICI therapy (149). Comparable to the preclinical biodistribution results, [^99m^Tc]Tc-tricarbonyl-NM-01 is rapidly excreted by the kidneys in humans, while both clinical studies revealed slightly higher uptake in the liver and bone marrow (147–149).

#### 3.1.4 PD-L1: K2

Broos et al. (150) discovered and evaluated K2 for its potential as both an imaging and therapeutic agent. Characterization of K2 was done alongside avelumab, and comparable nanomolar affinities toward PD-L1 were reported for both as measured by SPR (*K*_D_ = 3.8 and 1.6 nM, respectively). Furthermore, competition and dose-response SPR assays confirmed that K2 and avelumab share a PD-L1 binding epitope (150). In this study, K2 was radiolabeled with technetium-99m and evaluated in healthy mice and mice bearing breast cancer and melanoma tumors (150). Remarkably, SPECT/CT imaging in healthy mice revealed some of the lowest ever reported kidney uptake for Nb compared to a non-specific control Nb (150). In both cancer models, [^99m^Tc]Tc-tricarbonyl-K2 tumor uptake and tumor-to-blood ratios were sufficiently high, resulting in high contrast images at 80 min p.i. (150). It was further shown that K2 could activate TCR signaling normally inhibited by the PD-L1/PD-1 interaction and restore tumor cell killing in a shorter period than avelumab (150). The promising *in vitro* and *in vivo* results prompted this group to further optimize K2 as an imaging agent toward clinical implementation. First, the production of a gallium-68 labeled K2, [^68^Ga]Ga-NOTA-(hPD-L1), was explored by comparing two NOTA conjugation strategies (151). Since lysine is present in the CDR of K2, Bridoux et al. (151) determined to what extent the standard method, random conjugation of NOTA via free lysines, would influence the binding of K2 to PD-L1. Therefore, a sortase-A-mediated transpeptidation was explored as a strategy for site-specific conjugation of NOTA to K2 and was compared to the standard method (151). SPR affinity analysis resulted in highly similar *K*_D_ values for NOTA-K2 conjugated by lysine- and sortase-A-mediated methods (*K*_D_ = 3.7 and 4.41 nM, respectively) (151). Besides lower kidney uptake of the tracer with site-specific NOTA conjugation, PET imaging, and biodistribution results did not reveal any other differences between the two conjugation strategies (151). To ensure a reproducible and homogenously radiolabeled Nb-based tracer while avoiding the potential complications of enzymatic chelation procedure during clinical translation, another site-specific modification strategy using a maleimide (mal)-NOTA chelator was pursued (152). The affinity of NOTA-mal-(hPD-L1), as confirmed by SPR was not altered (*K*_D_ = 4.4 nM) (152). While [^68^Ga]Ga-NOTA-mal(hPD-L1) tumor uptake was high and comparable to the previous studies, kidney uptake was considerably increased compared with the other two conjugation strategies. K2 radiolabeled with gallium-68 shows promise as a diagnostic and therapeutic imaging agent targeting PD-L1, however, it is evident from these studies that the conjugation strategy can have an important effect on the kidney metabolism of Nbs in general.

#### 3.1.5 PD-L1: APN09

Was discovered by Ma et al. (153) and further developed as a gallium-68 PET imaging tracer. APN09 was conjugated to tris(hydroxypyridinone), or THP, by maleimide-Cys chemistry and subsequently radiolabeled with gallium-68 to form [^68^Ga]Ga-THP-APN09 (153). [^68^Ga]Ga-THP-APN09 was tested *in vitro* and *in vivo* and has already been tested in patients with NSCLC in a small clinical translation study (153). The affinity of [^68^Ga]Ga-THP-APN09 to PD-L1-transfected A549 cells (*K*_D_ = 22 nM) was determined by a cellular uptake assay (153). Tumor uptake could be observed in mice bearing both A549-PD-L1 and H1975 tumors, while lower relative uptake was achieved for the latter (153). Like most other reported PD-L1-targeting Nbs, [^68^Ga]Ga-THP-APN09 rapidly clears from the blood and is retained in the kidneys, resulting in high uptake in this organ (153). The in-human study further demonstrated safety and low radiation dose upon injection with [^68^Ga]Ga-THP-APN09, with high kidney uptake and low accumulation in the liver (153). Taken together, this study demonstrated the potential of this gallium-68-labeled Nb tracer to detect PD-L1 expression in patients with NSCLC.

#### 3.1.6 PD-1 Nbs

As is the case for other binders targeting the PD-L1/PD-1 axis, the number of Nbs specifically targeting PD-1 is limited. However, a couple of Nbs have already emerged in recent years as candidates with the potential to be developed as either therapeutic or imaging agents (154). PD-1 Nb20 was identified by standard immunization of a camel with recombinant human PD-1 followed by phage display screening of the resulting library and selection of promising lead Nbs (154). The Nb with the highest affinity for PD-1, PD-1 Nb20, as measured by SPR (*K*_D_ = 0.2 nM), was then further evaluated in combination with dendritic cell/tumor-fused cell (DC/tumor-FC) vaccines as an approach to enhance the anti-tumor efficiency of cytotoxic T lymphocytes (154). In combination with DC/tumor-FCs, PD-1 Nb20 was able to activate CD8^+^ T cells and inhibit tumor growth in mice bearing HCC827, HepG2, or Tca8113 tumors synergistically (154). More recently, another PD-1-targeting Nb, anti-PD-1 Nb-Fc, was discovered and has only been evaluated as a therapeutic agent preclinically (155). This group constructed a Nb library by extracting RNA from the spleen of a naïve camel opposed to immunization with a recombinant PD-1 protein (155). RNA was reverse transcribed, and the resulting cDNA was used as a template to amplify the VHH genes by PCR amplification. The amplicons were then later transformed into a naïve phage library, which was used for screening against PD-1 protein to identify the most promising Nb binder (155). To avoid rapid clearance from circulation, the Nb was fused with Fc to generate anti-PD-1 Nb-Fc (155). SPR and competitive ELISA confirmed the affinity of anti-PD-1 Nb-Fc to PD-1 (*K*_D_ = 6.6 nM) and the blocking efficiency of the PD-L1/PD-1 interaction, respectively (155). Tumor growth was effectively inhibited in a xenograft mouse model of human colorectal cancer after treatment with anti-PD-1 Nb-Fc, however, further characterization and pharmacokinetic analysis of this Nb have not yet been explored. Overall, these anti-PD-1 Nb binders have demonstrated potential for the development of more advanced therapeutic and, perhaps also, imaging agents.

### 3.2 Monobodies

Monobodies, also known as Adnectins, form part of a group of molecular scaffold proteins based on domain type III of the 10th human fibronectin (10Fn3) (156). They are structurally comparable to the heavy chain variable domain of immunoglobulins and consist of an anti-parallel β-sheet sandwich (156). Their target-binding properties are primarily attributed to the shared similarity between the diversified loops connecting the two β-sheets situated on opposite poles of 10Fn3 and the CDRs within immunoglobulin variable domains (57). Sequences present in these loops, similar to Abs and Nbs, can be subjected to diversification, enabling screening by display technologies (57). Due to the lack of Cys residues in their sequence, monobodies do not require linking disulfide bridges between β-sheets for proper folding and stability, providing them with more favorable thermodynamic properties and enhanced structural integrity (57). Favorable biophysical properties, especially high-affinity binding ability, make monobodies ideal candidates for therapeutic and imaging applications. Monobodies are small in size (approximately 94 amino acid residues) and can allow fast clearance of radioactively labeled tracers, quick imaging, and rapid penetration of solid tumors (157). Furthermore, a lysine residue is situated on the pole opposite to the binding region, allowing straightforward thiol- or amine-conjugation of chelators and radiolabeling (156). To date, only a handful of monobodies have been developed as PET imaging tracers to assess PD-L1/PD-1 expression and dynamics (158).

#### 3.2.1 PD-L1: BMS-986192

Was developed by BMS and has led to impressive results in multiple preclinical and clinical studies (41, 158–162). BMS-986192 was selected from a high complexity library using mRNA display, followed by adding a C-terminal Cys residue located on the opposite pole of the loops responsible for PD-L1 binding (158). This Cys enabled the conjugation of a suitable prosthetic group and radiofluorination to produce [^18^F]F-BMS-986192 (158). SPR measurements resulted in remarkably high affinities of both the unmodified and nonradioactive formats (BMS-986192) to human and cynomolgus PD-L1 (all *K*_D_ values < 10 pM) (158). High contrast PET images could be obtained 90-120 min p.i. of [^18^F]F-BMS-986192 in mice bearing PD-L1-expressing L2987 xenograft tumors (82). High kidney accumulation was evident of renal clearance of [^18^F]F-BMS-986192 (158). In a first-in-human study by Niemeijer et al. (41), the safety and feasibility of using [^18^F]F-BMS-986192 to predict therapy response in patients with NSCLC were evaluated. Although limited by the sample size of this study, a positive correlation between SUV_peak_ of [^18^F]F-BMS-986192 and therapy response of individual lesions could be made (41). To further investigate its potential in therapy response prediction, [^18^F]F-BMS-986192 was evaluated *in vitro*, by flow cytometry and Western blotting, and *in vivo*, by PET imaging of xenograft mice models bearing using human tumors with varying PD-L1 expression levels (lung mucoepidermoid carcinoma H292, lung adenocarcinoma H358, and ovarian clear cell carcinoma ES2 cell lines) (159). For both *in vitro* cell uptake and *in vivo* PET imaging, up- and downregulation of PD-L1 expression was achieved by treatment with IFNγ and selumetinib, respectively (159). Interestingly, while the treatment-induced change in PD-L1 expression was successfully achieved *in vitro*, PD-L1 expression levels remained unchanged in mice treated with IFNγ or selumetinib (159). Nevertheless, [^18^F]F-BMS-986192 demonstrated PD-L1 levels accurately in both scenarios (159). In a clinical pilot study, [^18^F]F-BMS-986192 PET/CT was used to determine PD-L1 expression in brain metastases of melanoma patients at baseline and in response to ICI (161). Similar to what Niemeijer et al. (41) demonstrated in NSCLC patients, [^18^F]F-BMS-986192 PET/CT could predict treatment response based on individual intracerebral and extracerebral lesions of melanoma patients (161). Besides the kidneys, other tissues that showed higher [^18^F]F-BMS-986192 uptake were the liver, spleen, and bone marrow (161). In an attempt to further facilitate the clinical translation of BMS-986192, radiolabeling with gallium-68 was explored (160). DOTA-maleimide was conjugated to the C-terminal Cys residue and subsequently radiolabeled with gallium-68 to form [^68^Ga]Ga-DOTA-BMS-986192 (160). PD-L1-transfected U-698-M B-cell lymphoma cells were tested *in vitro* and used to produce a tumor xenograft mouse model for *in vivo* tests (160). Cellular competition assays confirmed that [^68^Ga]Ga-DOTA-BMS-986192 binds to PD-L1 with high affinity (IC_50_ = 2.0 nM) (160). The *in vivo* results were highly comparable to [^18^F]F-BMS-986192, showing renal clearance and sufficient tumor to background signal less than 10 min p.i. (160). Zhou et al. (162) further explored an optimized gallium-68 labeled BMS-986192 using the more thermodynamically stable chelator NODAGA. Tumor uptake of [^68^Ga]Ga-NODAGA-BMS-986192 was rapidly visualized at 1 h p.i. and a quicker decrease in kidney uptake at 120 min p.i. compared to [^68^Ga]Ga-DOTA-BMS-986192 was notable (160, 162).

#### 3.2.2 PD-L1: FN3_hPD–L1_

FN3_hPD–L1_ was selected as a lead binder after screening a 10Fn3 yeast surface display library against recombinant human PD-L1, and its affinity was determined by BLI (*K*_D_ = 0.6 nM) (163). FN3_hPD–L1_ was evaluated further, and binding to cell-surface PD-L1 expressed on hPD-L1-transfected CT26 mouse colon carcinoma cells in culture was confirmed (164). In addition, NOD/SCID gamma (NSG) mice bearing CT26/hPD-L1 tumors were injected with His-tagged FN3_hPD–L1_, tumors were excised and stained (164). Both *in vitro* and *ex vivo* experiments demonstrated a sub-nanomolar affinity for this PD-L1 binder (164). A DOTA-NHS chelator was conjugated to the lysine group of FN3_hPD–L1_ and subsequently radiolabeled with copper-64 to form [^64^Cu]Cu-DOTA-FN3_hPD–L1_ (163). The tracer was tested *in vivo* in NSG mice bearing either murine (CT26/hPD-L1 mouse colon carcinoma) or human (MDA-MB-231 TNBC) tumor cell lines (160, 163). PET/CT imaging of both tumor models revealed rapid tumor uptake and good tumor-to-muscle ratio as early as 1 h p.i., which persisted up to 24 h p.i. (163). While uptake in the kidneys and the liver was higher due to clearance and metabolism, the tracer was rapidly cleared from other organs and tissue, resulting in high-contrast images (163).

### 3.3 Affibodies

A receptor, designated staphylococcal protein A (SPA), that is commonly found in the cell wall of *S. aureus* naturally binds to the Fc region of IgG and consists of five homologous domains (EDABC) (165). Domain Z is a modified analog of the SPA B domain, characterized by a helix bundle structure containing three α-helices and differs from its parent domain by a Gly29Ala substitution of helix two to increase stability (165). This domain forms the structural basis for another engineered scaffold protein, namely, an affibody (166). Unlike other binding scaffolds that make use of CDRs to introduce variability, the target binding residues of affibodies are located within solvent-exposed surfaces along two of the three α-helices and yield a flat-surface binding architecture (167). Moreover, the randomization of residues in the binding region of affibodies to obtain highly diverse combinatorial libraries is slightly more challenging, since the non-binding residues on the opposite side of the α-helix need to remain constant to preserve structural integrity and stability of the protein (166). Nevertheless, highly diverse combinatorial affibody libraries have successfully been constructed by randomization of 13 residues located on the solvent-exposed surface of helix one and two of the bundle (167). Affibodies consist of only 58 amino acid residues (7 kDa), rendering them a promising tool for imaging cancer-associated targets *in vivo* (167). Affibodies are further characterized by high proteolytic, chemical and thermal stability, a short folding time of 3 μs independent of disulfide bridge formation and are water-soluble (168–170). Affibodies are also suitable for solid-phase peptide synthesis, allowing the introduction of a handle at the N-terminus to incorporate various labeling moieties (171). Due to the absence of a cysteine residue in the polypeptide chain of an affibody, a cysteine can be introduced in the protein to allow site-directed conjugation of chelators, linkers and prosthetic groups using thiol chemistry (172, 173). Combined, these properties allow affibodies to perform well as molecular imaging tracers and therapeutic agents, with many demonstrating impressive results in preclinical and clinical studies (174–177).

#### 3.3.1 PD-L1: Z_PD–L1_

A lead affibody, labeled Z_PD–L1_1_, targeting PD-L1 was selected from phage display screening and has demonstrated promising *in vitro* and *in vivo* results as a PET tracer (178). A unique Cys residue engineered in the structure of Z_PD–L1_1_ was used to couple a NOTA chelator to yield NOTA-Z_PD–L1_1_ and binds to human and rhesus PD-L1 with affinities of *K*_D_ = 1.3 and 1.4 nM, respectively, as measured by SPR (178). NOTA-Z_PD–L1_1_ was fluorinated and evaluated in SCID Beige mice bearing either PD-L1-positive LOX malignant melanoma or PD-L1-negative SUDHL6 lymphoma tumors (178). Al[^18^F]F-NOTA-Z_PD–L1_1_ uptake could be visualized in PD-L1-positive tumors and was eightfold higher compared to PD-L1-negative tumors (178). Blood clearance was fast and tumor uptake in other organs remained low, except for the kidneys, with a very high uptake between of 254–312 %ID/g (178). The same group explored the use of an affinity-matured affibody, labeled Z_PD–L1_4_, to investigate whether higher affinity would lead to an improved *in vivo* targeting of PD-L1 in the same tumor model (174). Additionally, to assess biodistribution and endogenous PD-L1 targeting, the tracer was injected into healthy rhesus monkeys (174). In this study, NOTA-Z_PD–L1_4_ was radiolabeled with fluorine-18 and gallium-68 and injected tumor mouse models and healthy monkeys (174). SPR measurements confirmed an increased affinity (*K*_D_ = 70 pM) for NOTA-Z_PD–L1_4_. While [^68^Ga]Ga-NOTA-Z_PD–L1_4_ showed higher levels in the blood and plasma compared to Al[^18^F]F-NOTA-Z_PD–L1_4_, both tracers accumulated in PD-L1-positive tumors at levels 25-fold higher compared to PD-L1-negative tumors (174). Tissues known to express endogenous PD-L1, such as the lymph nodes and spleen, could also be visualized after injecting both tracers into healthy rhesus monkeys. Given the promising preclinical results from these studies, Sharma et al. (179) recently explored the use of Z_PD–L1_ radioconjugates to assess PD-L1 expression in subcutaneous and intracranial glioblastoma tumor models. Both [^68^Ga]Ga-NOTA-Z_PD–L1_ and Al[^18^F]F-NOTA-Z_PD–L1_ were rapidly taken up by tumors 1 h p.i. and led to high contrast tumor-to-brain parenchyma images (179).

#### 3.3.2 PD-L1: PDA

This affibody was developed by Liang et al. (180) and labeled with ^99m^Tc and used as a SPECT imaging tracer. A GGGC chelator was introduced into the sequence of PDA to facilitate radiolabeling with ^99m^Tc. Additionally, a hydrophilic HEHEHE-tag was incorporated in the sequence primarily for affibody recovery purposes during production, but could also assist in reducing liver retention (180). Affinity, specificity, and cellular internalization of [^99m^Tc]Tc-PDA were evaluated using mouse colon cancer cells transfected with human PD-L1 (MC38-B7H1) and wild-type cells (MC38) as control (180). [^99m^Tc]Tc-PDA could bind with high specificity and affinity to MC38-B7H1 cells (*K*_D_ = 10 nM), and ∼25% was internalized after 24 h (180). [^99m^Tc]Tc-PDA SPECT imaging was performed in C57BL/6J mice bearing MC38-B7H1 and MC38 tumors. Biodistribution revealed peak kidney uptake was reached 30 min p.i., while peak tumor uptake and tumor-to-tissue ratios were reached 120 min p.i. (180). Similar to Z_PD–L1_, [^99m^Tc]Tc-PDA was rapidly cleared from circulation and could detect PD-L1 expression in a short time frame (180).

#### 3.3.3 PD-L1: Z-j1 and Z-j2

While the preclinical results for the affibodies described above have proved promising thus far, little is still known about any protein-protein interaction or structural properties that contribute to their binding mode. Jing et al. (181) attempted to address this topic by using the yeast two-hybrid system (Y2H), a technique used to identify protein-protein interactions by screening a combinatorial library against a “bait” protein of interest, which in this case was human PD-L1 (182). Two promising affibodies, Z-j1 and Z-j2, were identified and selected as lead binders (181). While both affibodies could bind PD-L1, Z-j2 demonstrated superior inhibition of the blocking of the PD-1/PD-L1 interaction (181). Sequencing of the affibody library used for screening and lead selection revealed that amino acid mutations predominantly occurred in the first helix, with little to no variation occurring in the second and third helices (181). This finding provided important insight into the structure-activity relationship of affibodies with PD-L1 indicating that only a specific region of a binder could be involved in binding (181).

## 4 Peptides

**FIGURE 2:**
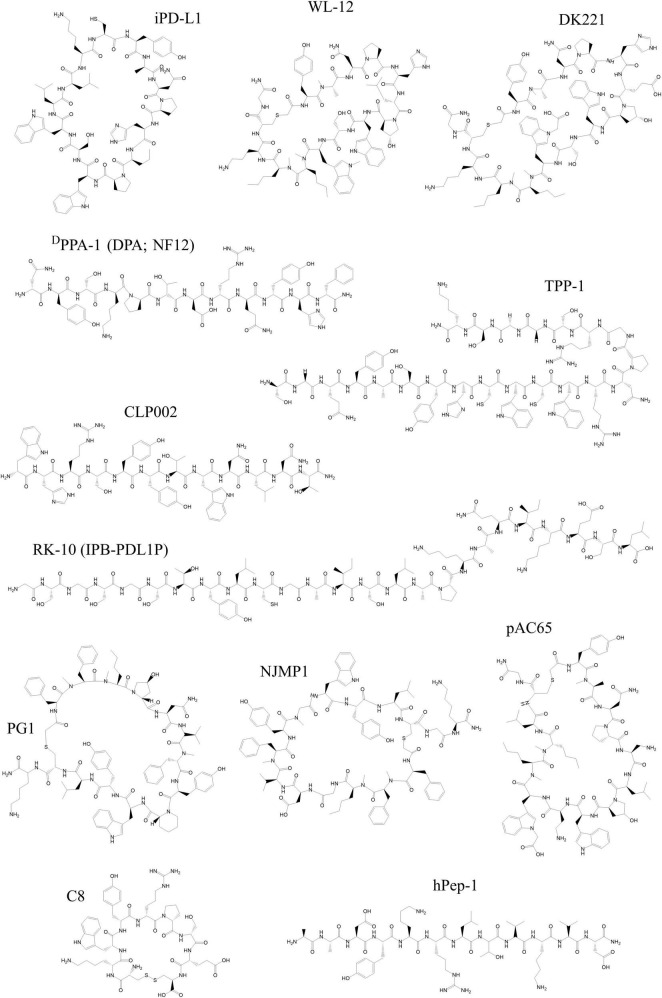
Chemical structures of peptides developed as tracers targeting PD-1 and PD-L1 discussed in this review. All structures were reproduced using the structural formula as provided in the respective reference indicated in the following list. The following peptides target PD-L1: WL-12 (191), DK221 (201), CLP002 (205), ^D^PPA-1 (DPA or NF12) (208), TPP-1 (211), RK-10 (IPB-PDL1P) (213), PG1 (215), NJMP1 (214), and pAC65 (216). The following peptides target PD-1: hPep-1 (219) and C8 (220). All structures were drawn using ChemDraw^®^.

Within the past decade, interest in using peptides as therapeutics and imaging tracers targeting the PD-L1/PD-1 axis has grown considerably. The list of peptides that target PD-L1 is continuously increasing. While many of these emerging peptides are being investigated as inhibitory drugs, the list of those developed as radiotracers is also growing (33). Peptides developed for imaging applications have slightly different criteria than those intended for targeted therapy. An extended half-life is often a desired property for the latter to maintain a sufficient concentration in circulation (183). Peptides developed as imaging tracers can have a shorter half-life and should ideally closely match the half-life of the radioisotope used (184). Radioisotopes that can be used for the radiolabeling of peptides include gallium-68, copper-64, zirconium-89, yttrium-90, technetium-99m, and indium-111 for indirect labeling via chelators (185). Direct labeling of peptides with or without a specific prosthetic group is also possible with fluorine-18, iodine-124, iodine-131, bromine-76, and astatine-211 (185). However, the most routinely used radioisotopes for labeling peptides in (pre-)clinical research are gallium-68, copper-64, and fluorine-18. Most likely this is due to their suitable half-lives, ease of production and compatibility with peptide chemistry (185). In addition to their more favorable features in the context of radiotracer development, such as small size, low immunogenicity and cost-effective production, peptides can easily be modified site-specifically for conjugation with linkers, chelators or other prosthetic groups (184). However, for the majority, the purpose of development is a therapeutic application or inhibition of the interaction with PD-1. Advanced chelating and radiolabeling approaches have already been established for peptides and have been reviewed extensively elsewhere (45, 186, 187). The following section will describe some of the most promising emerging peptides targeting the PD-L1/PD-1 axis from the past decade (see Figure 2 for a depiction of the structures of the peptides). The majority of peptides target PD-L1, as not many candidates have yet been developed against PD-1 (see Table 3).

**TABLE 3:** Programmed cell death ligand 1 and PD-1 binding peptides in development as radiotracers.

Target	Peptide (other names)	Format	Design strategy/ screening technique	Radiotracers	Affinity to human PD-L1/PD-1 (method)	Development stage	Docking/ crystallization
PD-L1	WL-12	Cyclic, 14 aa^a^	Selected from patented library (US9308236B2) (188)	[^64^Cu]Cu-DOTAGA-WL-12 (189, 190) [^64^Cu]Cu-NOTA-WL-12 (193) [^68^Ga]Ga-DOTAGA-WL12 (191) [^68^Ga]Ga-NOTA-WL12 (195) [^68^Ga]Ga-HBED-CC-WL12 (196) [^68^Ga]Ga-TRAP-WL12 (194)	[^nat^Cu]Cu-NOTA-WL12: *K*_D_ = 3.0 nM (SPR) (193)	Clinical (195)	Docking (189, 190)
	iPD-L1	Cyclic, 14 aa	Structure based design and molecular docking	[^99m^Tc]Tc-HYNIC-iPD-L1	iPD-L1: *Binding energy* = −6.7 kcal/mol (molecular docking) HYNIC-iPD-L1: *Binding energy* = −7.2 kcal/mol (molecular docking)	Clinical	Docking
	DK221 (DK222, DK223, based on WL-12)	Cyclic, 14 aa	Modified WL-12 (198)	Al[^18^F]F-NODA-DK222 (198–200) [^68^Ga]Ga-DOTA-DK221 (201)	DK222: *K*_D_ = 290 nM (BLI) (198) ^19^F-NODA-DK221: *K*_D_ = 530 nM (BLI) (198) DK221-DOTA: *K*_D_ = 1.0 nM (SPR) (201) DK221-DOTAGA: *K*_D_ = 5.7 nM (SPR) (201)	Preclinical	n.d.^b^
	CLP002 (CLP-2, HKP2201, HKP2202)	Linear, 12 aa	Phage display (202)	[^64^Cu]Cu-DOTA-HK2201 (205) [^68^Ga]Ga-DOTA-HK2201 (205)	CLP002: *K*_D_ = 366 nM (SPR) (202) [^68^Ga]Ga-DOTA-HK2201 (HK2202): *K*_i_ = 56 nM (cellular assay) (205) [^68^Ga]Ga-DOTA-HK2201 dimer (HK2203): *K*_i_ = 51 nM (cellular assay) (205)	Preclinical	Docking (202)
	^D^PPA-1 (DPA, NF12)	Linear, 12 aa	Mirror image phage display	[^64^Cu]Cu-DOTA-DPA (208) [^68^Ga]Ga-DOTA-DPA (208) [Al^18^F]F-NOTA-NF12 (209)	^D^PPA-1: *K*_D_ = 0.5 μ M (SPR) and 1.0 μ M (MST) (206) [^64^Cu]Cu-DOTA-DPA: *K*_i_ = 119 nM (cellular assay) (208) [^68^Ga]Ga-DOTA-DPA: *K*_i_ = 44 nM (cellular assay) (208) [Al^18^F]F-NOTA-NF12: *K*_D_ = 85 nM (cellular assay) (209)	Clinical (209)	Docking (209)
	TPP-1	Linear, 22 aa	Bacterial surface display	[^64^Cu]Cu-NOTA-TPP-1 (211) [^64^Cu]Cu-NOTA-PEG-TPP-1 (211) [Al^18^F]F-NOTA-TPP-1 (211) [Al^18^F]F-NOTA-PEG-TPP-1 (211)	TPP-1: *K*_D_ = 95 nM (SPR) (210) TPP-1 and TPP-1-NOTA: *K*_D_ ≈ 100 nM (SPR) (211)	Preclinical	Docking (210)
	IPB-PDL1P (RK-10)	Linear, 25 aa	Computational modeling (212)	Al[^18^F]F-NOTA-IPB-PDL1P (213)	n.d.	Preclinical	n.d.
	NJMP1 (based on BMS-78 and BMS-71)	Cyclic, 14 aa	Selected from patented library (US9308236B2) (188)	[^68^Ga]Ga-DOTA-NJMP1 (214)	NJMP1: IC_50_ = 242 nM (HTRF) (214) [^nat^Ga]Ga-DOTA-NJMP1: IC_50_ = 26 μ M (HTRF) (214)	Preclinical	n.d.
	PG-1 (based on BMS-78 and BMS-71)	Cyclic	Selected from patented library (US9308236B2) (188)	[^68^Ga]Ga-DOTA-PG1	DOTA-PG1: *K*_D_ = 10 nM (BLI) (215)	Preclinical	Docking (215)
	pAC65	Cyclic, 15 aa	Selected from patented library (US9308236B2) (188)	n.a.^c^	pAC65: IC_50_ = 1.8 nM (HTRF) (216)	*In vitro* characterization	Docking (216)
PD-1	**C8**	Cyclic, 7 aa	Phage display (220)	n.a.	C8: ***K*_D_ = 0.6 μ M** (220)	Preclinical	Docking (220)
	**hPep-1**	Linear, 12 aa	Interface peptide	[^64^Cu]Cu-DOTA-hPep-1	hPep-1: **EC_50_ = 136 nM** (luciferase blockade assay) hPep-1-DOTA: **EC_50_ = 170 nM** (luciferase blockade assay)	Preclinical	n.d.

^a^Amino acids; ^b^Not determined; ^c^Not applicable. These are the affinity values (in nanomolar) and crystallization resolution (in Ångström), they are written in bold for emphasis and quick identification.

### 4.1 PD-L1: WL-12

In 2014 BMS disclosed a library containing three classes of macrocyclic peptide inhibitors of the PD-L1/PD-1 interaction that sparked tremendous interest in developing these binders as both therapeutic and imaging agents (188). A promising PD-L1-targeting peptide selected from this disclosure that has gone through the most phases of development up to now is the highly specific 14-mer cyclic peptide WL-12. Chatterjee et al. have already explored the use of WL-12 as a potential PET tracer in the earliest stages of development by conjugating it with DOTAGA and radiolabeling with copper-64 (189). The IC_50_ of the metalated version of WL-12-DOTAGA was measured as 2.9 nM by a FRET-based assay (189). PET imaging of mice bearing human PD-L1 expressing CHO tumors could show rapid and specific uptake of the [^64^Cu]Cu-DOTAGA-WL-12 (14.9 ± 0.8 %ID/g 60 min p.i. in tumors) (189). To further obtain insight into the binding mode of WL-12 with PD-L1, docking studies were also performed. Overlays of PD-1 and WL-12 bound to PD-L1 revealed that WL-12 forms a beta-sheet comparable to a beta-sheet structure found within the interaction surface of PD-1 (189). The use of [^64^Cu]Cu-DOTAGA-WL-12 as a tool to evaluate the dynamic expression of tumor PD-L1 and target engagement of therapeutic anti-PD-L1 mAbs was successfully demonstrated (190). The same group further explored using gallium-68 (*t*_1/2_ = 67.7 min) with a half-life more closely matching the peptide pharmacokinetics (191). While tumor uptake was comparable for both versions of radiolabeled WL-12, [^68^Ga]Ga-DOTAGA-WL-12 resulted in faster blood clearance and improved imaging contrast but higher kidney uptake and lower liver uptake, compared to [^64^Cu]Cu-DOTAGA-WL-12 in the same tumor model (191). To ensure the feasibility of clinical translation and due to its easy access, the group explored an additional commonly used radioisotope, fluorine-18, for labeling of WL-12 (192). The ability of the native WL-12 and its non-radioactive analog (FPy-WL-12) to block the PD-L1/PD-1 interaction was measured by FRET-based assays, and EC_50_ values of 26.4 and 37.1 nM were reported, respectively (192). Compared to the previously developed labeled versions of WL-12, [^18^F]FPy-WL-12 exhibited lower tumor uptake and higher uptake in normal tissues, especially in the liver, which the group attributed to the influence of hydrophilicity and low labeling molar activity (192). Another group pursued this peptide further by investigating the effect of using a NOTA chelator instead of DOTAGA (193). The affinity of the non-radioactive [^nat^Cu]Cu-NOTA-WL-12 to human PD-L1 was determined by SPR (*K*_D_ = 3.0 nM) and comparable to affinities previously reported (193). The group demonstrated that [^64^Cu]Cu-NOTA-WL-12 could be prepared with higher radiochemical yield and purity compared to the DOTA counterpart (189, 193). More recently, Quigley et al. (194) reported on preclinical PET imaging in low PD-L1-expressing MDA-MB-231 human breast carcinoma and H2009 human lung adenocarcinoma tumor xenograft murine models using WL-12 conjugated to another chelator – TRAP – and radiolabeled with gallium-68. Compared to [^68^Ga]Ga-DOTA-WL-12, uptake in non-target organs, especially the liver, was lower and blood clearance more rapid for [^68^Ga]Ga-TRAP-WL-12 (194). Recently, Zhou et al. (195) performed the first-in-human evaluation of [^68^Ga]Ga-NOTA-WL-12 in patients with NSCLC and demonstrated its safety and feasibility to be used as a companion diagnostic to quantify PD-L1 expression. In a small number of lung cancer patients, different tumor uptake levels of [^68^Ga]Ga-NOTA-WL-12 correlated well with two different therapy response outcomes even though for these two particular cases, the PD-L1 expression determined by IHC was the same (195). [^68^Ga]Ga-NOTA-WL-12 uptake in the liver increased substantially and shifted toward the small intestines after adding WL-12 blocking (195). The group continues exploring ways to optimize the biological properties of this peptide-derived radiotracer further by testing other chelators, such as HBED-CC (196). Besides the initial study performed by Chatterjee et al. (189), no further studies to elucidate structural features of the peptide have been conducted yet. This is most likely due to the fact that sufficient affinity of this peptide, as well as that of its modified analogs, has already been achieved.

### 4.2 PD-L1: iPD-L1

Recently Ferro-Flores et al. (197) used structure-based design and extensive docking studies to develop another cyclic peptide, iPD-L1, based on the structure of WL-12 for SPECT imaging of PD-L1. The methylation of four residues and thioester could be removed in addition to replacement of the ornithine with a lysine present in WL-12. These modifications in combination with the introduction of a HYNIC heterocycle could result in a more rigid peptide structure (197). HYNIC-iPD-L1 was radiolabeled with technitium-99m and the tracer evaluated both preclinically and clinically. Molecular docking software was used to estimate the affinity to PD-L1 of both iPD-L1 and HYNIC-iPD-L1 in terms of molecular binding energy scores (−6.7 and −7.2 kcal/mol, respectively) (197). Affinity estimations of iPD-L1 were more favorable compared to WL-12 and biodistribution studies in mice bearing human lung cancer tumors (HCC827) showed higher tumor uptake at 24 h p.i. of [^99m^Tc]Tc-iPD-L1 (5.65 %ID/g) compared to [^99m^Tc]Tc-WL-12 (3.21 %ID/g), however for [^99m^Tc]Tc-iPD-L1 activity in non-tumor tissues was higher as well (197). A patient with plantar malignant melanoma underwent [^99m^Tc]Tc-iPD-L1 SPECT/CT imaging and the tracer could in fact distinguish between lesions with and without PD-L1 expression (197). This study clearly demonstrated the potential of using this modified cyclic peptide for detecting PD-L1 expression by SPECT imaging.

### 4.3 PD-L1: DK221, DK222, and DK223

The same group of researchers who initially developed WL-12 designed a derivative thereof, defined as DK221. In this work, the research focus was shifted toward developing imaging agents that could not only be used for imaging PD-L1, but that could be used more specifically as a tool to assess the pharmacodynamic behavior of therapeutic mAbs and determine the accessible PD-L1 target levels within the tumor (198). To this end, WL-12 was modified by introducing additional carboxylate groups in the peptide: L6E and Trp(Me)10Trp(carboxymethyl) (198). The ornithine group was replaced with lysine for modification with the bifunctional chelator NCS-MP-NODA and subsequently radiolabeled with fluorine-18 to form Al[^18^F]F-NODA-DK222 (198). The non-radioactive form was able to inhibit the PD-1/PD-L1 interaction with an EC_50_ = 25 nM measured by FRET assays and *in vitro* cell binding assays using Al[^18^F]F-NODA-DK222 confirmed its specificity for binding to PD-L1 (198). PET imaging and biodistribution studies in NSG mice bearing high PD-L1-expressing triple-negative breast cancer human xenografts using Al[^18^F]F-NODA-DK222 resulted in high contrast images with good tumor uptake (13.4 ± 0.1 %ID/g 60 min p.i.), high accumulation in the kidneys (57.7 ± 0.5 %ID/g 60 min p.i.) and low accumulation in the liver (198). Al[^18^F]F-NODA-DK222 was then further used to assess the residence time, or target engagement, of different anti-PD-1 and -PD-L1 therapeutic mAbs at the tumor site during treatment. Interestingly, Al[^18^F]F-NODA-DK222 PET was able to successfully quantify changes in PD-L1 occupation levels in real-time and could further reveal differences in the pharmacological activity of different anti-PD-L1 mAbs (199). An optimized protocol for the radiosynthesis of Al[^18^F]F-NODA-DK222 was later established that met the requirements for current GMP that could swiftly facilitate in-human studies, clinical translation and commercial production of this tracer (200). With similar aims in mind but exploring different radiochemistry, the same group performed a study by conjugating DK221 with the chelator DOTA-NHS for radiolabeling with gallium-68 to form [^68^Ga]Ga-DOTA-DK223 (201). Results were highly comparable to those obtained with Al[^18^F]F-NODA-DK222, showcasing the broad applicability and potential of this peptide-based imaging probe (201).

### 4.4 PD-L1: CLP002 (HKP2201 and HKP2202)

Liu et al. (202) were able to identify a linear 12-mer peptide, CLP002, by phage display biopanning with an affinity determined by SPR in the mid-nanomolar range (*K*_D_ = 366 ± 150 nM) able to block both the human and murine PD-1/PD-L1 interaction efficiently. Molecular docking studies revealed that the interaction surface of the peptide with PD-L1, similar to WL-12, overlaps with that of PD-1, however, the specific amino acid interactions between CLP002 and PD-L1 responsible for binding were not described (202). An interesting finding from this study was that one of the leads tested, CLP001, did not share a significant interaction surface area with PD-1, confirmed by blocking studies that showed little to no blocking efficiency of the human PD-1/PD-L1 interaction using recombinant PD-L1 and PD-L1 positive cancer cells (202). The group further showed that CLP002 had better tumor penetration and similar tumor growth inhibition capabilities compared to an anti-PD-L1 antibody (202). A known characteristic often observed mainly for linear peptides, is poor stability *in vivo* due to their susceptibility to protease degradation (203). The same group explored ways to improve CLP002 characteristics by performing side-chain macrocyclization scanning (204). This technique entails the substitution of two amino acids in the peptide sequence with a cysteine to produce all possible corresponding cyclic derivatives of the peptide. They discovered two cyclic CLP002 derivatives, CP7 and CP12, that had improved blocking efficiency of the PD-1/PD-L1 interaction, serum stability and anti-tumor activity compared to the parent peptide (204). Despite its inferior *in vitro* and *in vivo* results, Zhang et al. (205) decided to pursue the linear format of CLP002 further for its potential as a radiotracer to image PD-L1 in a colon cancer mouse model. The DOTA-conjugated CLP002, defined now as HKP2201, was radiolabeled with both copper-64 and gallium-68. A dimerized version, [^68^Ga]Ga-DOTA-HKP2202, was tested alongside HKP2201 to determine whether peptide dimerization can improve tumor uptake (205). Both ^64^Cu- and ^68^Ga-labeled HKP2201 showed modest tumor uptake, however, a significant reduction in liver uptake was observed for [^68^Ga]Ga-DOTA-HKP2201, making it the preferred labeling strategy for HKP2202. [^68^Ga]Ga-DOTA-HKP2202 could improve tumor uptake significantly at 60 min p.i. compared to [^68^Ga]Ga-DOTA-HKP2201 while maintaining the reduced uptake in the liver (205).

### 4.5 PD-L1: ^D^PPA-1 (DPA and NF12)

The first D-peptide identified by mirror-image phage display (MIPD) was identified and characterized *in vitro* (206). MIPD uses a D-protein of interest, in this case, D-PD-L1, in combination with a phage display library containing L-peptides for biopanning to identify L-peptide binders (207). The next step is synthesizing the corresponding D-enantiomeric form of the L-peptide binder that would bind to the original L-protein (207). The rationale behind developing such a peptide binder was to circumvent the inherent limitation of small proteins or peptides that have poor *in vivo* stability due to their sensitivity to proteolytic degradation in serum (203). Chang et al. (206) identified a 12-mer peptide inhibitor of the PD-L1/PD-1 interaction, defined as ^D^PPA-1, that showed no degradation in 10% human serum within a period of 24 h and that could bind to human PD-L1 with a *K*_D_ of 0.51 μM as measured by SPR. In addition to excellent stability, ^D^PPA-1 injected into CT26 tumor-bearing mice reduced tumor growth and increased survival time (206). A Cy5.5-conjugated ^D^PPA-1 was subsequently used for near-infrared fluorescence imaging to assess its biodistribution in the same mouse tumor model. Interestingly, while ^D^PPA-1 showed good uptake in the tumor, the peptide accumulated substantially in the liver and kidneys, in addition to some accumulation in the stomach and lungs (206). Since little was known about the metabolic profiles of D-peptides and their potential, the same group set out to investigate using this peptide as a PET imaging tracer (208). ^D^PPA-1, now referred to as D-dodecapeptide antagonist or DPA, was modified by adding a PEG_3_ linker and DOTA chelator for radiolabeling with copper-64 and gallium-68 (208). [^64^Cu]Cu-DOTA-PEG_3_-DPA was injected into WT (C57BL/6J) mice to determine the normal distribution of the radiotracer, and rapid clearance (within 20 min) from all major organs, including the heart, kidneys, and liver, was achieved (208). Urine samples were also collected at different time points up to 2 h p.i. and no degraded [^64^Cu]Cu-DOTA-PEG_3_-DPA could be found (208). Both [^64^Cu]Cu-DOTA-PEG_3_-DPA and [^68^Ga]Ga-DOTA-PEG_3_-DPA were injected into mice bearing B16F10 mouse melanoma or human glioblastoma tumors. In both cases, modest and rapid tumor uptake 60 min p.i. was observed in addition to slow tumor clearance, fast clearance from other organs and elimination via the kidney-bladder system (208). No significant decrease in red blood cells, platelets, or hematocrit was observed following injection of [^64^Cu]Cu-DOTA-PEG_3_-DPA injection measured up to 30 days p.i. suggesting potentially low toxicity (208). Another group recognized the potential of this peptide as a PET tracer and developed a fluorine-18 labeled version, defined it as Al[^18^F]F-NOTA-NF12, and performed the first human evaluation of this PD-L1 peptide binder (209). To take the characterization of this peptide tracer a step further, Zhou et al. (209) performed molecular docking to get insight into the binding mode of NF12 to PD-L1. The prediction revealed that NF12 binds to four amino acids on PD-L1, three of which have previously been identified as hot spot residues (48, 195). Healthy volunteers and patients with either NSCLC or esophageal cancer injected with Al[^18^F]F-NOTA-NF12 showed no clinical adverse or pharmacological events, and the tracer was eliminated primarily by the renal-urinary system (209). Compared to [^68^Ga]Ga-NOTA-WL-12, the accumulation of Al[^18^F]F-NOTA-NF12 in other organs, such as the liver, was substantially decreased (195, 209). Tumor PD-L1 expression was efficiently detected by Al[^18^F]F-NOTA-NF12 within a clinically relevant timeframe. Moreover, fast clearance and low non-specific uptake resulted in high-contrast images (209).

### 4.6 PD-L1: TPP-1

Targeting PD-L1 peptide 1 (TPP-1) was identified by bacterial surface display and has shown to be highly specific for PD-L1 (210). TPP-1 could bind to PD-L1 with high affinity as determined by a single cycle kinetic SPR measurement (*K*_D_ = 95 nM) and exhibited good binding to tumor cell lines expressing PD-L1 (210). TPP-1 could not only block the PD-1/PD-L1 interaction, as determined by both ELISA and cell-based blocking assays, but could efficiently inhibit tumor growth mediated by T cell reactivation in a tumor xenograft mouse model (210). A prediction of the binding site of TPP-1 on PD-L1 was explored by the same group, and similar to WL-12, a beta-sheet-like secondary structure is formed that interacts in the same PD-L1 binding pocket as PD-1 (189, 210). Kuan et al. (211) further investigated the potential TPP-1 as a PET imaging tracer by directly comparing fluorine-18 and copper-64 labeling strategies. Additionally, a pegylated tetramer of TPP-1 was also radiolabeled and tested to ascertain whether the pharmacokinetics and biodistribution of the tracer could be improved (211). Pegylated tetramer tracers were more stable than their native counterparts, confirmed by serum stability tests and longer retention in circulation up to 2 h p.i. (211). They found that TPP-1 and its pegylated tetramer labeled with copper-64 could successfully detect PD-L1 at the tumor site of MDA-MB-231 tumor-bearing mice compared to the negligible signal detected using those labeled with fluorine-18 (211). While significant liver accumulation was observed for the ^64^Cu-labeled tracers, higher spleen uptake could be achieved in normal (C57BL/6J) mice compared to ^18^F-labeled tracers (211). While there is still room for improvement, this study successfully described a strategy that could potentially improve radiotracer behavior *in vivo*.

### 4.7 PD-L1: RK-10 (IPB-PDL1P)

A 25-mer peptide, RK-10, was identified by exploiting X-ray crystal structure data available for the PD-1/PD-L1 interaction and using computational techniques to determine which amino acid sequences could have high probabilities of forming interactions with PD-L1 in the binding region (212). Different levels of PD-L1 could easily be detected by biotinylated or dye-conjugated RK-10, as demonstrated by IHC, flow cytometry, and patient tissue immunofluorescent staining (212). Notably, the excellent *in vitro* results for RK-10 suggest a high-affinity peptide, however, the actual affinity has not been measured by any standard techniques. Sun et al. (213) recognized the potential of RK-10 as a PET imaging tracer to evaluate PD-L1 status in tumors. RK-10 was modified with NOTA, radiolabeled with fluorine-18 to form Al[^18^F]F-NOTA-IPB-PDL1P and injected into mice bearing human colon carcinoma (HCT116), human prostate carcinoma (PC3) or CHO-K1 tumors (213). *In vivo* stability of Al[^18^F]F-NOTA-IPB-PDL1P was tested in serum collected from the blood of injected mice and showed intact tracer up to 1 h after injection, which was reflected by the prolonged tumor retention seen in the PET scans up to 2 h p.i. (213). However, uptake of Al[^18^F]F-NOTA-IPB-PDL1P in non-specific organs was considerable, even 2 h p.i., in addition to evident liver metabolism of this tracer (213). While this peptide has potential as a PD-L1-targeting tracer, further characterization and optimization are required to improve *in vivo* properties.

### 4.8 PD-L1: emerging BMS related peptides (PG-1, NJMP1 and pAC65)

A couple of cyclic peptides have recently emerged as promising PD-L1 binders, some with the potential to be used as imaging tracers (214–216). Similar to how WL-12 and DK221 were developed, these peptides are also derived from the earlier disclosure of BMS describing macrocyclic peptides inhibiting the PD-L1/PD-1 interaction (188). Not too long after, a group published their findings on the structural binding mode of two representative cyclic BMS peptides with PD-L1, BMS-71, and BMS-57, with IC_50_ values of 7 and 9 nM measured by HTRF assays and reported by BMS, respectively (188, 217). Jouini et al. (214) used the BMS-71/PD-L1 crystal structure complex as a guide and identified the C-terminal Gly-NH_2_ as a potential site for modification with DOTA and subsequent radiolabeling with gallium-68 to form their radiotracer, defined as [^68^Ga]Ga-DOTA-NJMP1. However, due to complications with the synthesis of BMS-71, they opted for BMS-78 (reported IC_50_ = 14 nM), a peptide identical to BMS-71 aside from an unmethylated glycine in BMS-78 (188, 214). Unfortunately, cellular binding studies demonstrated the inability of [^68^Ga]Ga-DOTA-NJMP1 to bind to PD-L1 (214). Accordingly, a significant decrease in the affinity of radiolabeled NJMP1 was observed, and it was concluded that contrary to the original hypothesis, the site for modification had a major influence on the binding mode (214). In addition, this group found a discrepancy in the affinity for BMS-78 with a 10-fold decrease in the value reported (214). Soon after, another group reported an optimized version of BMS-71, PG1, with superior *in vitro* and *in vivo* behavior (215). It was hypothesized that the methyl group of Gly in BMS-71 is a preferred site for modification since molecular docking of both BMS-71 and PG1 on a tetrameric PD-L1 structure revealed it is directed away from the other three monomers (215). The high affinity of the precursor, DOTA-PG1, was confirmed by BLI assays (*K*_D_ = 9.9 nM) and rapid [^68^Ga]Ga-DOTA-PG1 uptake in PD-L1-expressing A375 cells (215). PET/CT imaging and biodistribution in A375-PD-L1 tumor-bearing mice demonstrated rapid and high uptake of [^68^Ga]Ga-DOTA-PG1 as soon as 30 min p.i. (5.01 ± 0.46 %ID/g) that is retained in the tumor up to 2 h p.i. (6.96 ± 1.02 %ID/g) (215). In contrast with ^68^Ga-labeled WL-12 and DK223, high liver uptake was indicative of [^68^Ga]Ga-DOTA-PG1 metabolism primarily by the hepatobiliary system (191, 201, 215). Another macrocyclic peptide, pAC65, to keep an eye on, has recently been reported by Rodriguez et al. (216) to have affinity and bioactivity equivalent to current FDA-approved anti-PD-L1 mAbs. Based on the BMS-57/PD-L1 crystal structure complex previously reported by the same group, six hydrophobic amino acids in the structure of BMS-57 were identified and replaced by more hydrophilic amino acids to form pAC65 (216). pAC65 showed no evidence of toxicity at concentrations tested, however Jurkat effector cells could effectively be activated by pAC65 in a dose-dependent manner in the presence of PD-L1-expressing cells (EC_50_ = 0.6 nM) similar to atezolizumab (EC_50_ = 0.1 nM) (216). Initial *in vitro* results are promising for pAC65, and its potential to be developed further as a peptide-based therapeutic or imaging agent has been established.

### 4.9 PD-1 peptides

While the number is limited, a couple of PD-1-targeting peptide inhibitors have recently been described (218–220). The exact interaction surface of PD-L1 and PD-1 was uncovered by X-ray crystallography some time ago (48). Based on these structures, Boohaker et al. (218) selected a stretch of highly conserved amino acid residues present in both human and murine PD-L1 (Gly120 to Asn131) that has been shown to interact with PD-1 and investigated the 12-mer interface peptide as a PD-1 inhibitor. Another group explored the potential of this interface peptide to be used as a PD-1 PET imaging tracer (219). Human and mouse interface peptides were defined as hPep-1 and mPep-1, respectively, with mPep-1 containing a Leu instead of a Val at position 128 in the sequence of PD-L1 (219). Both hPep-1 and mPep-1 were conjugated with DOTA at the N-terminal and subsequently radiolabeled with copper-64 (219). Both tracers were evaluated in mice bearing murine melanoma B16F10 tumors (characteristic of PD-1 overexpression on TILs) and human hepatocellular carcinoma Huh-7 tumors. No tumor uptake was observed in the Huh-7 tumor model, while mPep-1-^64^Cu was able to detect PD-1 as soon as 20 min p.i. in the B16F10 tumor model (219). A cyclic 7-mer peptide, C8, has been identified by phage display and was shown to bind to PD-1 with an affinity of 0.64μM as measured by microscale thermophoresis (MST) (220). It was further shown that C8 was able to block the PD-L1/PD-1 interaction, activate T cells to a similar extent as an anti-PD-1 mAb, and could inhibit the growth of CT26 and B16 tumors, the latter being a known anti-PD-1 mAb resistant tumor model (220). These promising *in vitro* results warrant further exploring C8 as an imaging agent to quantify PD-1 expression levels.

## 5 Discussion

Since the discovery of immune checkpoints and the breakthrough of immunotherapy, it is undeniable that a positive response to treatment in cancer patients is possible, however, a one-size-fits-all treatment option is not attainable. What is also clear from the small fraction of clinical and preclinical studies described in this review, is that PD-L1 and PD-1-blocking mAb treatment show great promise. However, it is still unclear which mechanisms are involved in complete response, partial response or no response to treatment. Currently, efforts are dedicated toward the development, assessment, and optimization of predictive biomarkers in order to find the optimal treatment regime on a per-patient basis (28). Tumor PD-L1 expression has long been recognized as a potential predictive biomarker to assess the response to immunotherapy (221). Results from multiple clinical trials have proven that a positive correlation can be drawn between PD-L1 expression above a certain threshold and response to ICI (91, 92, 222, 223). However, some studies have also shown that treatment benefits may also be achieved in patients with tumor PD-L1 expression below this threshold (70, 224–226). A major drawback of assessing PD-L1 status by IHC is the spatial and temporal heterogeneity expression between different tumor lesions and within the same tumor tissue obtained from patient biopsies (227, 228). Non-invasive PET imaging using tracers that can accurately and repeatedly determine the level of PD-L1 or PD-1 expression in a cancer patient is a valuable tool that can certainly help to realize the full potential and predictive power of PD-L1 and PD-1 as prognostic biomarkers (33, 36, 229, 230). However, imaging studies using mAb-based PET tracers to detect PD-L1 levels have provided further contradictory evidence regarding the predictive value of PD-L1 expression for treatment response (65). PET imaging in human patients using ^89^Zr-labeled durvalumab, nivolumab and pembrolizumab could demonstrate a positive correlation, while [^89^Zr]Zr-DFO-atezolizumab tumor uptake gave no prediction of treatment response (41, 42, 65, 66, 74). These PET imaging studies clearly demonstrate differences in pharmacokinetics and tumor uptake of mAb-based tracers, even though they were developed to bind to the same target (either PD-1 or PD-L1). It may be that the inconsistent results from these studies are due to the choice of tracer type, i.e., mAbs. Tracers that are based on smaller biologicals such as nanobodies, affibodies, monobodies, or peptides have the potential to more accurately detect the PD-L1/PD-1 expression landscape, which in turn could reveal the true predictive power of these targets as biomarkers for treatment response.

While mAbs offer advantages such as high target affinity, specificity, tolerance to chemical modification, and exceptional target engagement, their long biological half-life is an undesired feature for imaging. Longer circulation times can cause high background signal and accumulation in non-target organs (e.g., the liver). Moreover, mAbs are most often radiolabeled with zirconium-89, a radiometal that leads to high energy gamma emission of 907.89 KeV (231). While ^89^Zr-PET can offer high-resolution images due to its short positron range and emission of β^+^ rays with E_β+,ave_ = 396 keV, a typical clinical dose of a ^89^Zr-labeled mAb can range between 37 and 74 MBq which translates to a dose of 20–40 mSv to a patient (231). In comparison, PET imaging using peptides or smaller proteins radiolabeled with short lived isotopes (i.e., fluorine-18, carbon-11, or gallium-68) can offer two- to fourfold less dose in patients (231). Another important drawback of mAbs is their large size of ∼150 kDa, which can result in ineffective tumor penetration and ultimately inaccurate measurement of PD-1/PD-L1 expression levels when used as an imaging tracer (232). On the other hand, non-full length mAb binders such as nanobodies, monobodies, affibodies, and peptides are considerably smaller in size (8 Da–80 kDa) and can offer advantages such as superior tissue penetration ability, low immunogenicity, and reduced production cost making them attractive candidates as imaging tracers (233, 234). The use of tracers with a size below the kidney’s glomerular filtration cutoff of ∼50 kDa often results in high and retained uptake in the kidneys, which can contribute to the absorbed dose by a patient (153, 158). This is especially the case for Nb-based tracers (235). In the context of diagnostic PET imaging, nephrotoxicity is not necessarily a major concern, especially when tracers are labeled with short-lived radioisotopes. However, imaging of tumors within or in close proximity of the kidneys can prove to be challenging using these tracers. To this end, ways to circumvent renal retention have been explored for peptides and Nbs. Co-administration with cationic amino acids can effectively outcompete binding to the endocytic megalin receptor highly abundant in the renal proximal tubule, while the use of dose fractionation, radio-protectors, and mitigating agents have all led to decreased renal uptake (236–239). Moreover, a single His-tag often present in Nbs for purification purposes can contribute to the overall polarity of a Nb, and its removal can further reduce the retention of Nb-based tracers in the kidneys (151, 239). The study performed by Broos et al. (150) showed remarkably low kidney uptake of an anti-PD-L1 His-tagged Nb SPECT/CT tracer, [^99m^Tc]Tc-tricarbonyl-K2, compared to non-specific control Nb. There were no speculations made regarding why this Nb led to reduced kidney retention, however, a comparative structural analysis of K2 compared to similar Nbs could provide important information that can be used for optimization of existing tracers. Unlike peptides and Nbs, kidney retention of affibodies is independent of the megalin receptor and the use of non-residualizing radiohalogens, and careful selection of the position of prosthetic group conjugation on the affibody structure, could result in reduced kidney uptake (240). In monobody-based imaging studies, the kidneys were also reported as the dose-limiting organ (158, 163). Robu et al. (160) demonstrated that another way to achieve reduced renal uptake is by co-injection of an excess of unlabeled [^68^Ga]Ga-DOTA-BMS-986192.

High *in vivo* stability is an important requirement for an optimal PD-L1/PD-1 imaging tracer. While there is considerable analogy between the variable domains of intact mAbs and the target binding domains of monobodies and affibodies, the absence of a Cys-residue in their structure makes them highly stable and allows them to withstand high temperatures and chemical modifications (57, 169). In addition, the option to introduce a unique Cys-residue in the structure of a binder that can allow site-specific conjugation of linkers, chelators, or other prosthetic groups is a significant advantage from an imaging perspective (54). Both natural and synthetic targeting peptides are limited by their instability in serum or plasma and are prone to degradation by proteolytic enzymes before reaching their intended target (241). Some of these limitations can be overcome by introducing structure modifications that can effectively improve the stability, affinity and specificity of peptides. For example, Chang et al. (206) attempted to enhance the stability of a human PD-L1 binding peptide, ^D^PPA-1, and used mirror-image phage display to develop a peptide with D-amino acid configuration. Unlike the L-enantiomer, the resulting peptide was not degraded in 10% human serum after 24 h of incubation (206). Opposed to the naturally occurring L-amino acids, D-enantiomer peptides have the unique ability to resist the degradative activity of endogenous proteolytic enzymes, resulting in peptides with higher biostability (242). Macrocyclization is another approach explored by Fetse et al. (204) to improve peptide stability. Cyclization by introducing two Cys-residues into the sequence of linear CLP-2 (or CLP002) resulted in increased proteolytic resistance and enhanced antitumor activity in a CT26 tumor model (204). Interestingly, the retro-inverso isomer of CLP-2 resulted in a significant loss of PD-1/PD-L1 blocking efficiency (204).

Exploring the molecular basis of the binding interaction of PD-L1/PD-1 tracers with their respective targets in more detail could provide useful insights for ways to optimize their properties and behavior *in vivo*. Crystal structures of PD-1 and PD-L1 with therapeutic mAbs – and with each other – have provided invaluable information that could be used to explain the molecular mode of inhibition of ICIs and to determine the exact binding epitope of PD-L1/PD-1 and paratopes of mAbs (46, 48). The central CC′FG β-sheet of PD-L1 was identified as a key region of interaction shared among inhibitors (62). While there are clear differences in the angle of interaction with this β-sheet and mAb paratope residues, five key “hot-spot” residues present in PD-L1 (Tyr56, Glu58, Arg113, Met115 and Tyr123) interact with all anti-PD-L1 mAbs (62). Thanks to the crystal structure of the potent anti-PD-L1 Nb, KN035 (also known as envafolimab), in complex with PD-L1, it is now known that four of these “hot-spot” residues are also important contributors to the tight interaction with KN035 (133). Crystal structure analysis of mAbs has further revealed that all CDRs, except for CDR-L2, are involved in binding to PD-L1 (62, 75). It is clear from the development of these small non-mAb binders that a PD-L1 binding domain much smaller in size – consisting of only a couple of amino acid residues – can result in the same PD-1-blocking efficiency and high binding affinity that are observed for mAbs (133, 158, 178, 189). High affinity (picomolar to low-nanomolar) is considered an important prerequisite for an optimal imaging tracer. While they did not disclose the specific methods used to achieve an affibody with ∼18-fold higher affinity, González Trotter et al. (178) and Rubins et al. (174) could demonstrate enhanced PD-L1 detection in tumors using an affinity-matured NOTA-Z_PD–L1_. However, a couple of peptide binders with mid-nanomolar to micromolar affinities, as determined by initial characterization studies, have demonstrated sufficiency in blocking the PD-1/PD-L1 interaction (CLP002: *K*_D_ = 366 nM, ^D^PPA-1: *K*_D_ = 0.5 μM and C8: *K*_D_ = 0.6 μM) (202, 206, 220). Interestingly, higher affinities were later reported for the radiotracer counterparts of some of these peptides. An affinity of *K*_D_ = 85 nM was reported for Al[^18^F]F-NOTA-NF12, a PD-L1-binding tracer with the same peptide sequence as ^D^PPA-1 (209). Similarly, [^68^Ga]Ga-DOTA-HKP2201, which is the radiolabeled format of CLP002, could outcompete unlabeled HKP2201 in a PD-L1 competition binding assay with *K*_i_ = 56 nM (205). Besides affinity maturation, other methods such as tetramerization (for ^64^Cu- and ^18^F-labeled TPP-1) and dimerization (for ^64^Cu- and ^68^Ga-labeled HKP2202) have led to improved tumor uptake and biodistribution (205, 211).

Docking studies were performed for nearly all the peptide binders reviewed above and led to meaningful information regarding molecular mode of binding and potential optimization strategies. For example, docking studies of PG-1 (a macrocyclic peptide based on BMS-78 and BMS-71) and a PD-L1 tetramer could clearly pinpoint which residues present in PG-1 had the correct orientation suitable for DOTA conjugation (215). Moreover, docking of WL-12 to the co-crystal structure of PD-L1 and PD-1 revealed that WL-12 binds with a β-sheet-like structure that overlaps considerably with two β-sheets present in PD-1 responsible for interaction with PD-L1 (189). In contrast with the peptide binders of the PD-1/PD-L1 axis, limited structural information and docking studies are available for nanobodies, monobodies and affibodies. Besides, KN035, no other X-ray crystallization studies have been attempted for other Nbs (211). While most of the Nbs (and other radiotracers) described in this review were specifically developed to block the PD-L1 and PD-1 interaction, *in vitro* and *in vivo* studies have shown that [^68^Ga]Ga-NOTA-Nb109 and [^99m^Tc]Tc-tricarbonyl-NM-01 binds uncompetitively to a different PD-L1 epitope (141, 147). Considering that limited structural information is currently available for these binders, it would be worthwhile to solve the crystal structures of Nb109 and NM-01 in complex with PD-L1 to uncover additional “hot-spot” residues important for binding only and not inhibition. Besides the detection of temporal and spatial expression of PD-L1, an anti-PD-L1 peptide tracer, Al[^18^F]F-NODA-DK222, was designed to specifically evaluate PD-L1 occupation levels by mAbs in real-time instead (199). Differences in the pharmacological activity of the various anti-PD-L1 mAbs could be determined in this study (199). A radiotracer that can bind to PD-L1 or PD-1 with a different epitope could facilitate these types of investigations into therapeutic activity in addition to assessment of target expression levels while therapy is ongoing and target occupation (at the inhibitory binding epitope) is assumed. In turn, a better understanding of immunotherapy response could be obtained, and the predictive value of PD-L1 and PD-1 expression could be enhanced.

Thus far, development of non-mAb-based radiotracers specifically targeting PD-1 has been limited (30, 242). In the context of diagnostic imaging in oncology, targeting PD-L1 takes precedence due to the ability to visualize both the tumor and the heterogeneous expression of PD-L1, whereas PD-1 is primarily expressed on lymphocytes. Therefore, imaging of PD-1 is more representative of tumor infiltration by PD-1-expressing lymphocytes. Moreover, preclinical evaluation of PD-L1-targeting radiotracers is more straightforward in animal models bearing tumors overexpressing PD-L1. Out of the four non-mAb PD-1 binders addressed in this review, only hPep-1 has been explored preclinically as a PET tracer to detect PD-1 expression (208). Furthermore, the potential of pAC65, FN3_PD–L1_, and Z-j2 as PD-L1 binders has already been established, and development into PET imaging agents should be explored (164, 181, 216).

## 6 Conclusion

Positron emission tomography imaging using a suitable radiotracer to assess the dynamic expression landscape of PD-1 and PD-L1 could provide a better prediction of treatment response compared to the current FDA-approved evaluation by invasive biopsy and IHC. While great progress has been made toward finding an optimal PD-1/PD-L1 radiotracer, the optimal tracer that can be used routinely has not been identified yet. Optimizing a binder is a crucial step during the development phase and a clear understanding of the structural and molecular basis of the radiotracer-target interaction is imperative for optimizing physico-chemical properties to achieve high specificity of the PET signal in patients.

## Author contributions

MB: Conceptualization, Data curation, Writing – original draft. AW: Supervision, Writing – review & editing. WB: Supervision, Writing – review & editing.
